# Nanoimprinted Infrared Metasurface Multispectral Filters for Neural‐Network‐Assisted Low‐Temperature Sensing

**DOI:** 10.1002/nap2.70345

**Published:** 2026-11-02

**Authors:** Shiyu Yang, Zhijuan Su, Yaping Dan

**Affiliations:** ^1^ Global College Shanghai Jiao Tong University Shanghai China; ^2^ School of Integrated Circuits Shanghai Polytechnic University Shanghai China

**Keywords:** infrared metasurfaces, multispectral filters, nanoimprint lithography, neural networks, thermometry

## Abstract

Low‐temperature infrared sensing requires temperature information to be extracted from weak thermal radiation while preserving optical throughput. Nanoimprinted metasurface filters combine multispectral encoding with neural‐network calibration to address this challenge. A single circular‐ring geometry controls passband position, bandwidth, and overlap, with simulated tuning across 6.4–11.2 μm. Deep‐ultraviolet stepper lithography and ultraviolet nanoimprinting transfer reusable master patterns to 1‐inch zinc sulfide filters. Four broad, partially overlapping bands retain peak transmittance near 80% and provide complementary spectral inputs for blackbody measurements from −20°C to 20°C. Neural‐network regression on the processed calibration curves achieves a mean absolute error of 0.160°C, compared with 0.434°C for one unfiltered input, a reduction of approximately 63%. Temperature‐resolved comparisons also identify lower errors at the cold end of the range. By connecting geometric spectral design, replicable fabrication, and computational temperature retrieval, this approach offers a route to compact multispectral thermometers that make better use of weak infrared signals.

## Graphical abstract

Nanoimprinted ring‐aperture filters turn weak infrared radiation into four geometrically tuned spectral inputs. Array‐period control places broad, overlapping bands with peak transmittance near 80%. Neural‐network calibration retrieves −20°C to 20°C blackbody temperatures with a mean absolute error of 0.160°C, approximately 63% below a single unfiltered input.

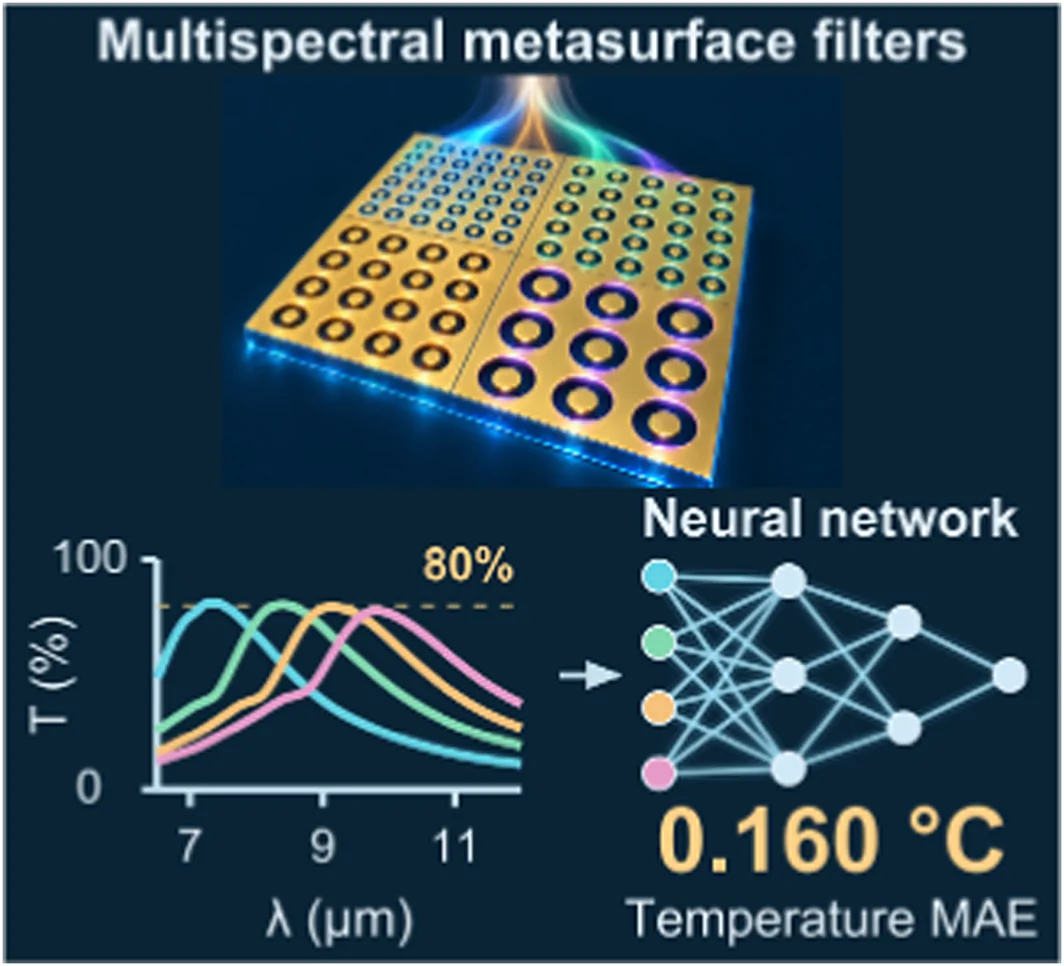

## 1 Introduction

Surface temperature is a primary observable of the polar energy budget. The temperatures of seawater, glacier ice, icebergs and sea ice reflect heat exchange among the ocean, atmosphere and cryosphere, and temperature anomalies near glacier fronts accompany dynamic processes such as accelerated melting, retreat and calving [1, 2, 3]. Sustained, spatially resolved temperature data therefore support both climate research and operational needs, including environmental monitoring and navigation safety. However, acquiring such data remains challenging. Satellite thermal infrared imagery offers broad spatial coverage but is limited in resolving rapid local variations by revisit intervals, cloud cover, atmospheric correction and ground sampling distance [4, 5, 6]. Shipborne and in situ instruments achieve high accuracy at fixed points, yet deployment is constrained by floating ice, unstable glacier fronts, severe weather and maintenance costs. Compact non‐contact sensors suitable for vessels, unmanned platforms and distributed stations would therefore complement both approaches [7, 8, 9].

Infrared thermometry infers temperature from received thermal radiance [10]. For an ideal blackbody, Planck's law gives

(1) $$B_{\lambda }\left(T\right)=\frac{2hc^{2}}{\lambda^{5}}{\left[\exp\left(\frac{hc}{\lambda k_{B}T}\right)-1\right]}^{-1},$$

where T is absolute temperature, λ the wavelength, h Planck's constant, c the speed of light and $k_{B}$ the Boltzmann constant. Equation (1) makes explicit that temperature governs not only the integrated emitted power but also the spectral shape of the emission. Real measurements sense that shape only through the response of the full optical chain. For the *i*th spectral channel the photocurrent can be written as

(2) $$I_{i}\left(T\right)=G_{i}\int B_{\lambda }\left(T\right)\varepsilon \left(\lambda \right)\tau_{\text{atm}\;}\left(\lambda \right)F_{i}\left(\lambda \right)R_{d}\left(\lambda \right)d\lambda +b_{i}.$$

Here, *F_i_(λ) i* is the filter transmission, *R_d_(λ)* is the detector responsivity, ε(λ) is the target emissivity, *τ*_atm_ (*λ*) is the atmospheric transmission, *G_i_* is the effective gain and optical coupling, and b_*i*_ is the background offset. Because *G_i_, ε* and *τ*_atm_ enter multiplicatively, an intensity change carries no unique signature of its origin: a shift in *I_i_* may reflect a temperature change, or equally a change in standoff distance, surface condition, atmospheric path or alignment. These effects motivate multispectral detection of low‐temperature targets.

Multispectral imaging supports applications including change detection, surface‐water monitoring and biomedical imaging [11, 12, 13, 14, 15]. For thermometry, multispectral detection can address this degeneracy. Acquiring several wavelength‐weighted measurements of the same scene, rather than one broadband integral, preserves information about spectral shape. Between −20°C and 20°C the blackbody emission maximum shifts from 11.5 to 9.9 μm, so channels placed in and around this interval sample both the temperature‐sensitive spectral slope and the high‐radiance region near the peak. Additional spectral channels can improve retrieval performance when the signal is jointly controlled by temperature and non‐temperature factors.

Translating this principle into a compact sensor is constrained by optical implementation. Grating spectrometers deliver high spectral resolution at the cost of long optical paths and tight alignment tolerances [16, 17, 18, 19]. Filter wheels acquire bands sequentially and introduce moving parts, which suits neither fast transients nor unattended deployment. Multilayer interference filters are mature, but integrating multiple bands at the pixel pitch over large areas demands intricate deposition, patterning and registration [20, 21, 22]. Metasurfaces offer an alternative in which the spectral response is determined by subwavelength in‐plane geometry rather than by stack thickness or beam path, so distinct passbands can be lithographically defined side by side in a single ultrathin layer [23, 24, 25, 26, 27]. Mid‐infrared plasmonic multispectral filters have demonstrated this capability for spectral selection [28, 29, 30, 31, 32].

The ring‐aperture design combines simple replication, broad thermal spectral weighting and an in‐plane symmetric geometry. Tan et al. use circular Al apertures for filter‐array chemical spectroscopy [33]; Kuruoglu et al. use oppositely tilted elliptical apertures in silicon membranes for thermally tunable molecular spectroscopy [34]; and Sulejman et al. use rectangular silicon resonators for polarization‐switchable near‐infrared microscopy [35]. These studies motivate a distinction between throughput‐oriented thermal encoding, actively tuned narrow resonances and polarization‐dependent image processing. Measured peak transmittance is compared in the Results and Discussion.

A deployable platform must nevertheless satisfy four coupled requirements: a filter geometry simple and reproducible enough for large‐area patterning; continuous tunability across the long‐wave infrared band relevant to cold targets; pixel‐level integration of multiple channels within a detector‐compatible footprint [36]; and a retrieval scheme capable of inverting the nonlinear coupling in Equation (2). Manufacturing is often the primary bottleneck. Electron‐beam lithography writes features serially, so exposure time scales with patterned area and resonator count. Nanoimprint lithography instead transfers an entire pattern in parallel, and once a master mold is qualified the same layout can be replicated repeatedly, amortizing the cost of the demanding step across many devices [37, 38, 39, 40]. For the retrieval stage, a neural network is well matched to learning the nonlinear map from a multidimensional current pattern to temperature, using reproducible interchannel differences that resist description by a single analytical calibration curve [41, 42, 43]. The network does not substitute for the physical mechanism: the metasurface array performs spectral encoding, and the network decodes it.

Here, nanoimprinted ring‐aperture filters connect infrared spectral design with neural‐network temperature retrieval. Two geometric parameters tune broad, overlapping passbands within one material stack, while reusable master patterns enable fabrication on 1‐inch ZnS substrates and a pixelated 2 × 2 prototype. The measured filter bands extend from approximately 7 μm to beyond 11 μm with peak transmittance near 80%. Their complementary responses enable four‐channel regression with a mean absolute error of 0.160°C over −20°C to 20°C, compared with 0.434°C for one unfiltered input in the processed‐calibration evaluation. The central advance is to combine substantial photon throughput with useful spectral diversity in a simple, replicable optical structure—a basis for compact computational thermometry of cold surfaces.

## 2 Results and Discussion

### 2.1 Multispectral Encoding of Low‐Temperature Radiation

Figure 1 shows a potential sensing concept, targeting non‐contact monitoring of seawater, glacier surfaces, floating ice and sea ice near or below the freezing point. A broadband detector integrates the incident spectrum into one scalar; optical throughput is high, but the wavelength dependence that distinguishes temperature from geometry and surface condition is discarded. Four spectral channels encode the source radiation as a current vector,

(3) $$\hat{I}\left(T\right)={\left[I_{1}\left(T\right),I_{2}\left(T\right),I_{3}\left(T\right),I_{4}\left(T\right)\right]}^{T},$$

where each component represents a different spectral weighting of the same source. Detection thus becomes compact multispectral encoding rather than one‐dimensional radiometry.

**FIGURE 1:**
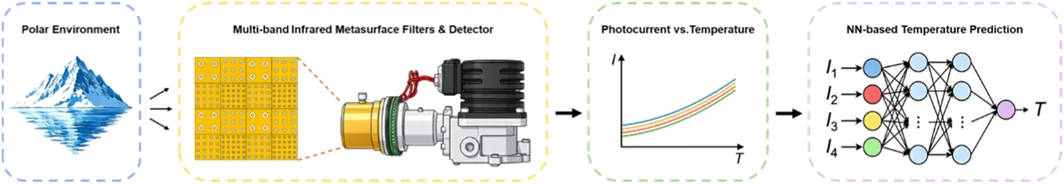
From thermal radiation to multispectral temperature retrieval. A conceptual scene of seawater, glacier surfaces, floating ice, and sea ice illustrates the motivation for non‐contact sensing of cold surfaces. Metasurface filters encode the incident radiation into complementary spectral channels, and a neural network combines their photocurrents to infer temperature. The pixelated arrangement illustrates a route toward compact thermal imaging; the photocurrent curves are qualitative sketches with similar rising trends and small interchannel differences, without an assigned numerical temperature or current scale.

The band placement follows the physics of Equation (1). Over −20°C to 20°C the emission maximum shifts from 11.5 to 9.9 μm; the filter set spans roughly from 7 μm to beyond 11 μm, covering both the short‐wavelength flank, where radiance has a larger fractional temperature dependence, and the high‐radiance region near the peak. The flank supplies sensitivity, the peak supplies signal, and sampling both balances the two.

Partial overlap between adjacent passbands is deliberate. Near‐identical responses would make channels redundant and add little information, whereas very narrow, widely separated bands would reduce throughput and leave gaps in the sampled spectrum. Controlled overlap allows neighboring channels to share part of the incident flux while retaining distinct spectral weights, which makes their relative responses informative. Broadband perturbations that act principally on *G*_*i*_, such as defocus or changes in standoff distance, shift all channels in a correlated way, whereas a temperature change alters the shape of *B*_*λ*_(T) and therefore changes the channels differentially. The vector I consequently encodes both overall magnitude and spectral distribution.

Each channel integrates radiation over wavelength, so spectral differences appear in the slopes, curvature, and relative changes of the photocurrents. The neural network combines these responses to identify the temperature‐dependent pattern across channels. Planar filter arrays also offer a compact optical format that can place spectral selection close to the detector, making the same encoding principle relevant to pixel‐level readout.

A 2 × 2 multispectral unit offers a route to pixel‐level temperature readout when aligned with a detector array. Its spatial‐sampling trade‐off and reconstruction options are discussed in Supporting Information S1: Section S7.

### 2.2 Geometrically Tunable Ring Metasurface Filters

The filter is a periodic array of annular apertures in a continuous Au film defined by period P, inner diameter $D_{1}$ and outer diameter $D_{2}$ (Figure 2a). We chose the ring because it supports a strong infrared resonance while remaining a single‐topology structure that can be scaled parametrically and replicated reliably. Under infrared illumination the ring sustains resonant charge oscillation with localized fields concentrated at its edges; the simulated near‐field distribution reaches $\left|E\right|/\left|E_{0}\right|=15$, showing localized electric‐field enhancement at the aperture edges. This resonance mediates transmission through the metal–dielectric stack and defines the passband.

**FIGURE 2:**
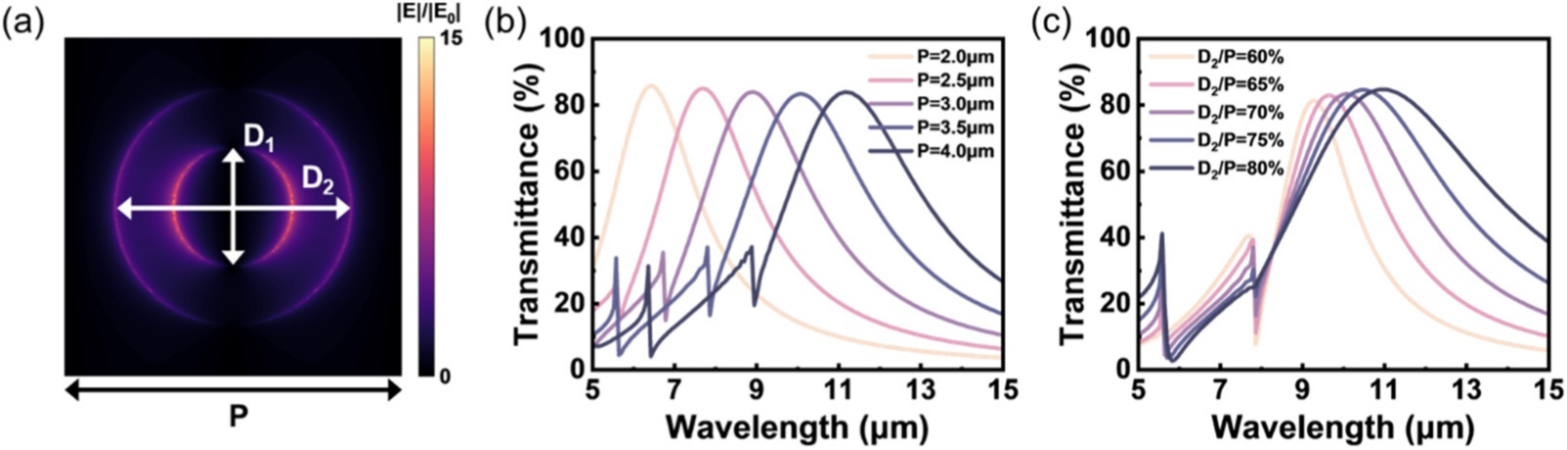
Geometric tuning of the ring metasurface bandpass filter. (a) Unit cell of the periodic annular‐aperture array in an Au film on ZnS, defined by array period P, inner diameter $D_{1}$ and outer diameter $D_{2}$, with the simulated near‐field amplitude $\left|E\right|/\left|E_{0}\right|$ at the resonance under normally incident illumination. Field enhancement reaches $\left|E\right|/\left|E_{0}\right|=15$ at the aperture edges, showing localized electric‐field enhancement. (b) Simulated transmission spectra for P increasing from 2.0 to 4.0 μm at fixed $D_{2}/P$, showing monotonic red‐shift of the passband across 6.4–11.2 μm. (c) Simulated transmission spectra for $D_{2}/P$ increasing from 60% to 80% at fixed P, broadening the full width at half maximum from approximately 2.2 to 6.2 μm. All spectra are finite‐difference time‐domain results at normal incidence.

Passband position is set by the array period (Figure 2b). Increasing P rescales the aperture geometry and lattice spacing, producing the simulated red‐shift of the transmission band. Varying P from 2.0 to 4.0 μm moves the passband monotonically from the mid‐ to the long‐wave infrared, covering 6.4–11.2 μm. Monotonic tuning gives a direct design rule: a target channel wavelength is reached by changing one lithographically defined in‐plane dimension, with no change to the material stack and no new resonator geometry.

Bandwidth is set by the normalized diameter $D_{2}/P$ (Figure 2c), which changes the aperture size relative to the period and the metal filling fraction. Smaller $D_{2}/P$ gives a narrower simulated passband; larger $D_{2}/P$ broadens the simulated response. Increasing $D_{2}/P$ from 60% to 80% widens the full width at half maximum from approximately 2.2 to 6.2 μm.

Together, Figure 2b,c establish a two‐parameter design space in which P places the band and $D_{2}/P$ sets its width and the overlap with its neighbors, without altering the ring topology. This is precisely the control the application needs. The objective is not four isolated narrowband filters but four complementary weightings of the same low‐temperature spectrum: too much overlap and the channels duplicate one another, too little and both throughput and spectral continuity suffer. The four selected designs distribute across the long‐wave infrared with distinct but partially overlapping bands, trading channel contrast against transmitted power.

Deriving all four channels from one structural platform has a practical consequence beyond design economy. A single material stack, imprint condition and etch recipe serve every channel, with the spectral differences encoded purely in local geometry. One master mold can therefore carry all four ring arrays and transfer them in a single imprint cycle, making the architecture as suited to parallel replication as it is to spectral tuning.

### 2.3 Nanoimprint Fabrication From a Wafer‐Scale Master

Figure 3 shows the fabrication route, designed to deliver nanoscale pattern fidelity and large‐area replication simultaneously. The distinction matters: one nanostructured field suffices to demonstrate a resonance, whereas a sensor requires many filter pixels with consistent geometry and spectral response.

**FIGURE 3:**
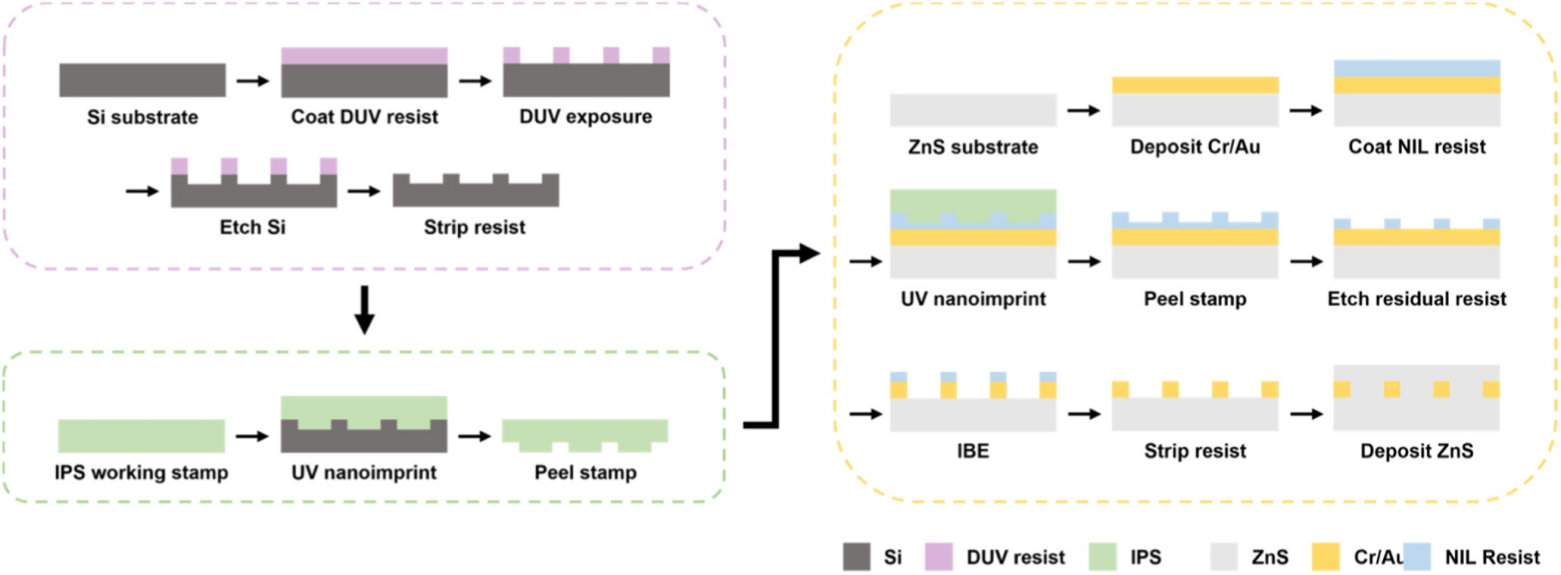
Nanoimprint fabrication route from an 8‐inch silicon master. Process flow for the pixelated metasurface filters. A silicon master mold is defined by deep‐ultraviolet (DUV) stepper lithography and etching, encoding both the nanoscale ring geometry and the multispectral pixel layout in a single body. An intermediate polymer stamp (IPS) working mold is replicated from the silicon master by UV nanoimprinting. On the device substrate, 3 nm Cr, 100 nm Au and a UV‐curable resist are deposited on a 1‐inch polished ZnS substrate; the stamp is brought into conformal contact, the resist is cured under UV exposure and released, the residual layer is removed, and the pattern is transferred into the Au film by Ar ion‐beam etching. Stripping the remaining resist and depositing a 300 nm ZnS overlayer completes the stack. All features within the stamp field transfer in parallel, and the master is reusable across successive stamps and devices.

Fabrication begins with a silicon master mold. Deep‐ultraviolet photoresist is spin‐coated on an 8‐inch silicon wafer and exposed by deep‐ultraviolet (DUV) stepper lithography, defining both the ring arrays and the pixel layout; the pattern is then etched into the silicon and the residual resist stripped. Repeated exposure of a compact mask design extends the periodic layout across the master; the stepper positioning precision is approximately 30 nm. Intermediate polymer stamp (IPS) working molds are replicated using an Eitre‐6 system with 6‐inch circular IPS films. The 8‐inch master is divided into four sectors to fit this system's 6‐inch loading capacity. The master relief is copied onto the IPS film before the working stamp transfers the patterned area to the resist‐coated ZnS substrate. The relatively rigid patterned IPS surface helps retain the ring features. Each 1‐inch ZnS device is patterned in a single imprint; the periodic arrangement is established by stepper lithography and transferred by nanoimprinting. Supporting Information S1: Section S1 gives the fabrication conditions, and Supporting Information S1: Section S6 provides process‐stage images.

Devices are built on ZnS for its infrared transparency across the band of interest. A 3 nm Cr adhesion layer and a 100 nm gold film are deposited on the ZnS substrate and overcoated with a UV‐curable imprint resist. The IPS working stamp is brought into contact, the pattern is fixed by UV curing, and after demolding the residual resist layer is removed. The pattern is transferred into the gold film by ion‐beam etching, chosen because gold is chemically inert and not efficiently patterned by conventional reactive‐plasma chemistries. The remaining resist is stripped and a 300 nm ZnS overlayer is deposited to passivate the patterned metal and complete the stack.

The fabrication route is chosen primarily to combine fine‐feature patterning with practical large‐area fabrication on ZnS. Conventional contact lithography is difficult for the submicrometer ring features, whereas electron‐beam lithography can resolve them but requires time‐consuming serial writing over the device area. DUV stepper lithography defines these features on a reusable silicon master. The available stepper is configured for large silicon wafers rather than the small ZnS optical substrates used here. Nanoimprinting therefore transfers the silicon‐master pattern onto a 1‐inch ZnS substrate, matching the detector window without requiring a costly larger ZnS substrate. Repeated stepper exposures extend a compact mask pattern across the master; the completed pattern is then copied in one imprint. Master reuse also supports manufacture of additional sensors, even when each sensor has a long service life.

### 2.4 Pixelated Arrays and Optical Characterization

Figure 4 presents the fabricated arrays and their measured spectra. Stepper‐lithography patterning of the filter layouts is shown in Figure 4a, and the 1‐inch ZnS device in Figure 4b. Figure 4c shows the pixelated architecture: each multispectral unit comprises four approximately 30 × 30 μm filter pixels in a 2 × 2 arrangement with approximately 1 μm gaps. The four designs correspond one‐to‐one to the spectral channels intended for detector pixels. The optical micrograph resolves the periodic pixel grid and the SEM inset resolves the four ring arrays. For spectral characterization and temperature tests, corresponding single‐pattern devices provide each band over the illuminated area, simplifying acquisition of the four spectral inputs with the available linear‐array detector. Figure 4d therefore characterizes the four constituent designs of the multispectral unit.

**FIGURE 4:**
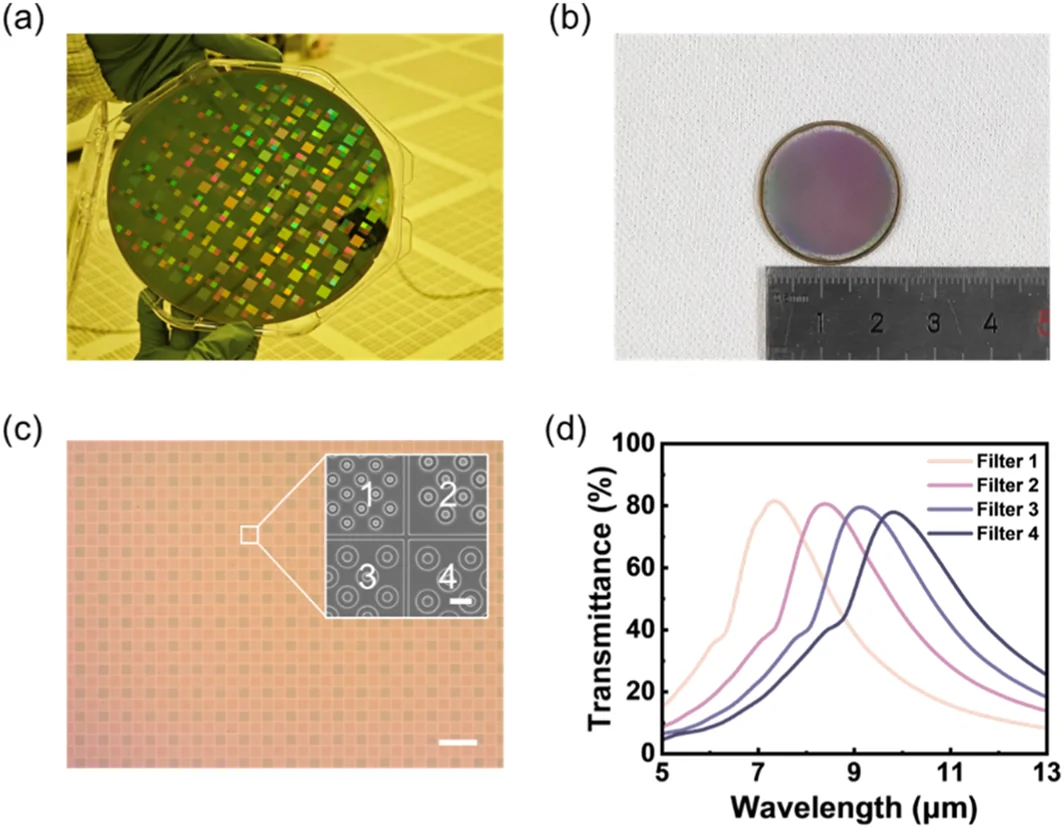
Pixelated filter arrays and measured spectral response. (a) DUV stepper‐lithography patterning of the filter layouts. (b) Completed 1‐inch‐diameter ZnS metasurface filter device. (c) Optical micrograph of the pixelated array; inset, SEM image of a 2 × 2 multispectral unit resolving the four ring arrays. Scale bars: 100 and 2 μm. (d) Transmission spectra of the four corresponding single‐pattern designs, acquired in transmission mode using a Thermo Scientific iN10 MX infrared microscope. Design numbering follows the inset. A bare ZnS substrate is measured as the reference, and the spectra include the single‐interface ZnS transmission correction described in Supporting Information S1: Section S2. The distinct, partially overlapping passbands extend from approximately 7 μm to beyond 11 μm and reach peak transmittance near 80%.

Measured transmission spectra of the four filters (Figure 4d) show distinct, partially overlapping passbands distributed from approximately 7 μm to beyond 11 μm, with peak transmittance approaching 80%. The quoted range includes passband tails; the four representative resonance peaks are 7.325, 8.390, 9.130 and 9.820 μm. Supporting Information S1: Figures S1 and S2 and Tables S1 and S2 present two measurement sets: representative spectra for simulation–measurement comparison and additional five‐position spectra for spatial characterization. The set therefore samples the short‐wavelength slope and the region approaching the emission maximum for blackbody temperatures between −20°C and 20°C, as intended. Rather than four isolated bands, the spectra form a continuous sequence of shifted weightings whose crossing points progress across the long‐wave infrared; the functional quantity is the collective organization of the four curves, not the exact crossover wavelength of any adjacent pair. This arrangement fixes the balance between redundancy and contrast: shared spectral regions correlate neighboring channels, while differences in band center and shape preserve channel‐specific weighting, so subtle changes in the thermal spectrum can be encoded without resorting to very narrow passbands.

Supporting Information S1: Figure S1 and Table S1 compare the simulated and measured spectra. The measured peaks are blue‐shifted by 0.030–0.175 μm, with peak transmittance lower by 2.19–5.95% points. Sloped aperture walls and a ZnS overlayer that follows the underlying steps, rather than the flat surface assumed in simulation, can affect resonance and transmission. Supporting Information S1: Section S2 discusses these structural differences and possible thin‐film effects in detail. The measured filters retain the broad, overlapping passbands needed for thermal spectral encoding.

Table 1 compares measured peak transmittance across five representative experimental infrared metasurface‐filter studies. Au hole arrays reach approximately 60% [32]; Ag micropipe arrays show peaks of approximately 25%–60% [44]; Al‐aperture arrays show peak levels around 40% [33]; and GeSbTe (GST)‐embedded plasmonic filters reach approximately 70% [45]. Hybrid Al–Ge filters report measured transmission above 60% [46]. The four filters here provide 77.91%–81.48% peak transmittance with broad, partially overlapping long‐wave infrared passbands. This combination retains optical throughput while supplying distinct spectral weightings for low‐temperature regression. Graphical estimates and measurement references are identified in the table; the comparison concerns reported peak throughput, with wavelengths and structures stated as context.

**TABLE 1:** Representative comparison of measured peak transmittance in experimental infrared metasurface filters.

Study	Measured peak T [%]	Structure/wavelength/measurement basis
Wang and Dan [32]	≈ 60	Au hole arrays on Ge; 5.3 μm example; bare‐Ge reference
Xu et al. [44]	≈ 25–60^a^	Ag micropipes; resonances near 5–11 μm; transmitted/incident intensity for each array
Tan et al. [33]	≈ 40^a^	Al apertures on Si; 41‐channel FTIR measurement
Julian et al. [45]	≈ 70	GST‐embedded Ag nanoholes; measured FTIR resonance near 2.9–3.35 μm
Soliman et al. [46]	> 60	Al–Ge passband near 2.6 μm; bare‐CaF_2_ reference
This work	77.91–81.48	Four ZnS‐filter spectra; peaks 7.325–9.820 μm; single‐interface correction

*Note:* Approximate values read from published figures are marked as graphical estimates; the measurement basis is retained for each study. These reported values describe throughput under the respective measurement conditions rather than a common renormalized efficiency.

^a^ Graphical estimate from the published experimental plot; values are approximate and are not digitized raw‐data maxima.

### 2.5 Temperature‐Dependent Detector Response and Neural‐Network Prediction

Blackbody measurements from −20°C to 20°C in 0.1°C steps provide one unfiltered broadband response and four filtered responses (Figure 5a). The unfiltered channel produces the largest current and steepest rise with temperature. The filtered channels lie within approximately 1.22–1.30 μA but differ in relative variation. Four 1‐inch single‐pattern filters are read sequentially along the same optical path at each setpoint, with the filtered readout averaged over four detector pixels. The infrared linear‐array detector operates at a fixed bias of 0.2 V. Supporting Information S1: Section S3 and Figure S5 describe the optical setup, temperature conditions, and processing; Supporting Information S1: Section S6 gives the relative detector response.

**FIGURE 5:**
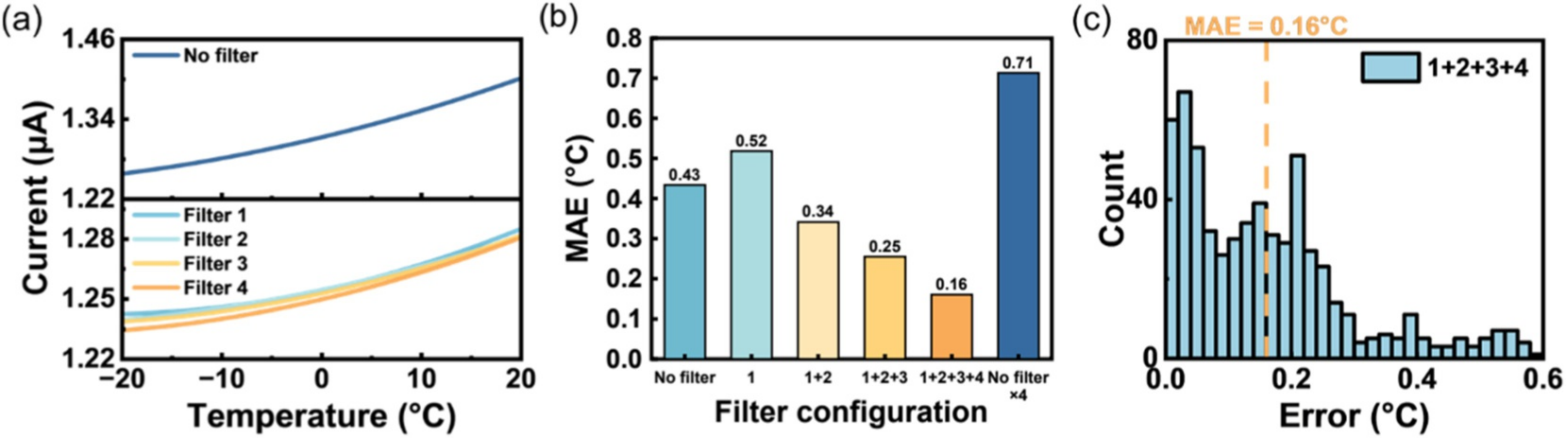
Detector response and neural‐network temperature retrieval. (a) Measured photocurrent versus blackbody temperature from −20°C to 20°C for the unfiltered aperture and the four filtered channels. The unfiltered channel gives the largest current and steepest slope; the four filtered channels lie within 1.22–1.30 μA, a span of 0.08 μA, and differ in slope and relative variation rather than in absolute magnitude. (b) Mean absolute error of the retrieved temperature for different input configurations: unfiltered broadband (0.43°C), one filtered channel (0.52°C), two channels (0.34°C), three channels (0.25°C) and the full four‐channel input (0.16°C). The two‐filter input is *F*1 + *F*2. The four‐unfiltered‐pixel control gives 0.71°C; Supporting Information S1: Table S4 provides the complete metrics. (c) Absolute‐error distribution for the four‐filter configuration; the dashed line indicates MAE = 0.16°C. The errors are calculated relative to the blackbody target temperatures.

Similar current magnitudes can carry different spectral information. Equation (2) weights the blackbody spectrum by each filter transmission and the detector response. A channel emphasizing the short‐wavelength side therefore changes differently with temperature from one emphasizing the emission peak. Across the scan, the four currents form a joint temperature‐dependent pattern that the network can calibrate. The value of an added channel depends on how its temperature response complements the other inputs and on the noise shared between them; the local information model and correlated‐noise limit are given in Supporting Information S1: Section S4.

The current curves were averaged, smoothed by a local polynomial Savitzky–Golay procedure and interpolated onto a uniform 0.01°C grid before regression. Averaging and smoothing reduce random readout fluctuations and estimate the smoothly varying current–temperature trend; interpolation aligns the channel inputs at common target temperatures. The acquisition, processing settings and evaluation on processed calibration curves are detailed in Supporting Information S1: Section S3 and Statistical Analysis. The network learns

(4) $$\hat{T}=f_{\theta }\left(I_{1},I_{2},\text{…},I_{m}\right),$$

where *m* is the number of input channels, θ the trainable parameters and $\hat{T}$ the predicted temperature. It acts as a nonlinear calibration that absorbs the combined effects of thermal emission, filter transmission, detector responsivity and system response.

Figure 5b compares six input configurations in the same processed‐calibration evaluation. The unfiltered broadband input gives a mean absolute error (MAE) of 0.43°C. Among the filtered configurations, the MAE decreases from 0.52°C for one channel to 0.34°C for two, 0.25°C for three, and 0.16°C for four. To assess whether multiple broadband inputs alone reproduce the benefit of spectral encoding, four separately measured unfiltered pixel currents are used as a four‐input control, giving an MAE of 0.71°C. Thus, the four‐filter configuration combines useful spectral inputs with the lowest MAE among these tested configurations; its MAE is approximately $\frac{1}{2.7}$ of that of the single broadband input. The acquisition definitions and matched target grid are specified in Supporting Information S1: Section S3.

For the four‐filter regression configuration (Figure 5c), MAE = 0.160°C, RMSE = 0.2090°C, *R* ^2^ = 0.9997 and maximum absolute error = 0.5858°C. Supporting Information S1: Figure S4 gives predictions and signed residuals for the same 601 target–prediction pairs. Supporting Information S1: Figure S6 and Table S5 resolve MAE by blackbody target temperature in fixed 5°C intervals. In −20 to −15°C, four‐filter MAE is 0.455°C, compared with 0.974°C and 1.927°C for one and four unfiltered inputs, respectively. All eight intervals and their point counts are reported. These temperature‐resolved results identify the cold‐end benefit within the processed‐calibration evaluation.

At low target temperatures, weaker thermal emission and smaller current changes per degree make temperature estimation more sensitive to readout fluctuations. The filters emphasize different parts of the spectrum, and their relative responses change with temperature even when the absolute currents are similar. The network combines these changes to distinguish nearby temperatures. In the coldest interval, MAE is reduced by 53.3% relative to one unfiltered input and 76.4% relative to four unfiltered inputs. This comparison highlights the value of combining optical spectral encoding with computational calibration for weak thermal signals.

## 3 Conclusion

Nanoimprinted ring‐aperture metasurfaces link high‐throughput infrared filtering with computational temperature retrieval. Geometric tuning controls passband position and overlap within a single material stack, while master replication provides a practical route to 1‐inch ZnS devices and pixelated filter layouts. Four measured bands spanning approximately 7 μm to beyond 11 μm retain peak transmittance near 80%. In the processed blackbody calibration from −20°C to 20°C, four‐channel regression reaches 0.160°C MAE, approximately 63% lower than the single unfiltered input, with a pronounced benefit in the coldest interval. The significance lies in combining photon collection, complementary spectral responses, and neural‐network inference in an optical structure that is simple to tune and replicate. This combination provides a foundation for compact low‐temperature thermometers and for extending multispectral temperature readout to pixelated sensing systems.

## 4 Methods

Experimental measurements. The 1‐inch ZnS devices use 3 nm Cr/100 nm Au and a 300 nm ZnS overlayer. Spectra are measured relative to bare ZnS using a Thermo Scientific iN10 MX microscope; the reported spectra include the single‐interface correction defined in Supporting Information S1: Section S2. Five sampled positions per design characterize local variation. Four filters are measured sequentially in a six‐position wheel at each blackbody setpoint. Supporting Information S1: Sections S1–S3 provide fabrication parameters, the coated‐device cross section, optical distances and data processing. ZnS dispersion is based on Debenham [47]; the modeled spectral range and substrate‐interface convention are specified in Supporting Information S1: Section S2.

## 5 Statistical Analysis

Spectral summaries use mean ± sample standard deviation across five sampled locations per design (*n* = 5; denominator *n* − 1). Regression MAE, RMSE and *R* ^2^ are calculated from all 601 exported target–prediction pairs per configuration. Temperature‐dependent MAE uses fixed 5°C bins with counts in Supporting Information S1: Table S5. These counts describe the processed test grid. Input and target standardization use the full processed dataset before the random training/validation/test split, as detailed in Supporting Information S1: Section S3. Analysis uses Python with NumPy, pandas and openpyxl; the regression model uses PyTorch and scikit‐learn.

## Author Contributions

**Shiyu Yang:** methodology, investigation, data curation, formal analysis, writing – original draft, visualization. **Zhijuan Su:** funding acquisition, investigation, project administration, supervision, writing – review and editing. **Yaping Dan:** conceptualization, methodology, validation, formal analysis, supervision, funding acquisition, writing – review and editing.

## Conflicts of Interest

The authors declare no conflicts of interest.

## Supporting information


Supporting Information S1

## Data Availability

The data supporting the findings of this study are available from the corresponding authors upon reasonable request.

## References

[1] N. Wunderling , M. Willeit , J. F. Donges , and R. Winkelmann , “Global Warming Due to Loss of Large Ice Masses and Arctic Summer Sea Ice,” Nature Communications 11, no. 1 (2020): 5177, 10.1038/s41467-020-18934-3. PMC7591863 33110092

[2] T. Kanzow , A. Humbert , T. Mölg , et al., “The System of Atmosphere, Land, Ice and Ocean in the Region Near the 79N Glacier in Northeast Greenland: Synthesis and Key Findings From the Greenland Ice Sheet–Ocean Interaction (GROCE) Experiment,” Cryosphere 19, no. 5 (2025): 1789–1824, 10.5194/tc-19-1789-2025.

[3] T. Li , S. Hofer , G. Moholdt , et al., “Pervasive Glacier Retreats Across Svalbard from 1985 to 2023,” Nature Communications 16, no. 1 (2025): 705, 10.1038/s41467-025-55948-1. PMC11735618 39814715

[4] P. Reiners , J. Sobrino , and C. Kuenzer , “Satellite‐Derived Land Surface Temperature Dynamics in the Context of Global Change—A Review,” Remote Sensing 15, no. 7 (2023): 1857, 10.3390/rs15071857.

[5] A. Seward , S. Ashraf , R. Reeves , and C. Bromley , “Improved Environmental Monitoring of Surface Geothermal Features Through Comparisons of Thermal Infrared, Satellite Remote Sensing and Terrestrial Calorimetry,” Geothermics 73 (2018): 60–73, 10.1016/j.geothermics.2018.01.007.

[6] S. Chen , H. Ren , X. Ye , J. Dong , and Y. Zheng , “Geometry and Adjacency Effects in Urban Land Surface Temperature Retrieval From High‐spatial‐resolution Thermal Infrared Images,” Remote Sensing of Environment 262 (2021): 112518, 10.1016/j.rse.2021.112518.

[7] J. Rietz , S. T. S. van Beeck Calkoen , N. Ferry , et al., “Drone‐Based Thermal Imaging in the Detection of Wildlife Carcasses and Disease Management,” Transboundary and Emerging Diseases 2023 (2023): 5517000, 10.1155/2023/5517000. PMC12017128 40303672

[8] S. J. Dugdale , J. Klaus , and D. M. Hannah , “Looking to the Skies: Realising the Combined Potential of Drones and Thermal Infrared Imagery to Advance Hydrological Process Understanding in Headwaters,” Water Resources Research 58, no. 2 (2022): e2021WR031168, 10.1029/2021wr031168.

[9] A. E. McKellar , N. G. Shephard , and D. Chabot , “Dual Visible‐Thermal Camera Approach Facilitates Drone Surveys of Colonial Marshbirds,” Remote Sensing in Ecology and Conservation 7, no. 2 (2021): 214–226, 10.1002/rse2.183.

[10] T. Tanaka , K. Miyazaki , and T. Yabuki , “Observation of Heat Transfer Mechanisms in Saturated Pool Boiling of Water by High‐Speed Infrared Thermometry,” International Journal of Heat and Mass Transfer 170 (2021): 121006, 10.1016/j.ijheatmasstransfer.2021.121006.

[11] E. J. Parelius , “A Review of Deep‐Learning Methods for Change Detection in Multispectral Remote Sensing Images,” Remote Sensing 15, no. 8 (2023): 2092, 10.3390/rs15082092.

[12] D. R. Panuju , D. J. Paull , and A. L. Griffin , “Change Detection Techniques Based on Multispectral Images for Investigating Land Cover Dynamics,” Remote Sensing 12, no. 11 (2020): 1781, 10.3390/rs12111781.

[13] C. Albertini , A. Gioia , V. Iacobellis , and S. Manfreda , “Detection of Surface Water and Floods With Multispectral Satellites,” Remote Sensing 14, no. 23 (2022): 6005, 10.3390/rs14236005.

[14] Q. Fang and Z. Wang , “Cross‐Modality Attentive Feature Fusion for Object Detection in Multispectral Remote Sensing Imagery,” Pattern Recognition 130 (2022): 108786, 10.1016/j.patcog.2022.108786.

[15] F. Ma , M. Yuan , and I. Kozak , “Multispectral Imaging: Review of Current Applications,” Survey of Ophthalmology 68, no. 5 (2023): 889–904, 10.1016/j.survophthal.2023.06.004. 37321478

[16] S. Mukhtar , A. Arbabi , and J. Viegas , “Compact Spectral Imaging: A Review of Miniaturized and Integrated Systems,” Laser & Photonics Reviews 19, no. 21 (2025): e01042, 10.1002/lpor.202501042.

[17] P. Kaufmann , H. M. Chrzanowski , A. Vanselow , and S. Ramelow , “Mid‐IR Spectroscopy With NIR Grating Spectrometers,” Optics Express 30, no. 4 (2022): 5926–5936, 10.1364/oe.442411. 35209544

[18] R. Haïdar , G. Vincent , S. Collin , et al., “Free‐Standing Subwavelength Metallic Gratings for Snapshot Multispectral Imaging,” Applied Physics Letters 96, no. 22 (2010): 221104, 10.1063/1.3442487.

[19] E. Sakat , G. Vincent , P. Ghenuche , et al., “Guided Mode Resonance in Subwavelength Metallodielectric Free‐Standing Grating for Bandpass Filtering,” Optics Letters 36, no. 16 (2011): 3054–3056, 10.1364/ol.36.003054. 21847157

[20] A. Jiang , O. Yoshie , and L. Chen , “Multilayer Optical Thin Film Design With Deep Q Learning,” Scientific Reports 10, no. 1 (2020): 12780, 10.1038/s41598-020-69754-w. PMC7392768 32728183

[21] B. Qin , H. Zhu , R. Zhu , M. Zhao , M. Qiu , and Q. Li , “Space‐To‐Ground Infrared Camouflage With Radiative Heat Dissipation,” Light: Science & Applications 14, no. 1 (2025): 137, 10.1038/s41377-025-01824-y. PMC11947227 40140379

[22] M. Bembenek , M. Makoviichuk , I. Shatskyi , et al., “Optical and Mechanical Properties of Layered Infrared Interference Filters,” Sensors 22, no. 21 (2022): 8105, 10.3390/s22218105. PMC9659176 36365803

[23] X. Zou , R. Lin , Y. Fu , et al., “Advanced Optical Imaging Based on Metasurfaces,” Advanced Optical Materials 12, no. 6 (2024): 2203149, 10.1002/adom.202203149.

[24] H. Zhou , C. Zhao , C. He , L. Huang , T. Man , and Y. Wan , “Optical Computing Metasurfaces: Applications and Advances,” Nanophotonics 13, no. 4 (2024): 419–441, 10.1515/nanoph-2023-0871. PMC11501951 39635656

[25] X. Zhao , T. Lou , Y. Peng , F. Sun , and X. Wei , “Metasurface‐Based Photodetectors: Pursuing Superior Performance and Multifunctionality,” ACS Photonics 12, no. 8 (2025): 4096–4118, 10.1021/acsphotonics.5c00691.

[26] C. Zhang , L. Cong , J. Zhang , et al., “Advanced Metasurfaces: Precise Modulations of Light in Space and Time Domains,” Advanced Photonics 8, no. 1 (2026): 014003, 10.1117/1.ap.8.1.014003.

[27] D. Wang , Z. Liu , H. Wang , M. Li , L. J. Guo , and C. Zhang , “Structural Color Generation: From Layered Thin Films to Optical Metasurfaces,” Nanophotonics 12, no. 6 (2023): 1019–1081, 10.1515/nanoph-2022-0063. PMC11501295 39634932

[28] Q. Zheng , Li Liang , S. Yang , et al., “Broadband on‐chip Spectral Sensing via Directly Integrated Narrowband Plasmonic Filters for Computational Multispectral Imaging,” Nanophotonics 14, no. 27 (2025): 4957–4964, 10.1515/nanoph-2025-0398. PMC12717906 41426096

[29] J. Yang , K. Cui , Y. Huang , W. Zhang , X. Feng , and F. Liu , “Angle‐Insensitive Spectral Imaging Based on Topology‐Optimized Plasmonic Metasurfaces,” Laser & Photonics Reviews 18, no. 12 (2024): 2400255, 10.1002/lpor.202400255.

[30] S. Rosas , S. K. Biswas , W. Adi , et al., “Mass‐Manufactured Gradient Plasmonic Metasurfaces for Enhanced Mid‐IR Spectrochemical Analysis of Complex Biofluids,” Advanced Materials 37, no. 47 (2025): e04355, 10.1002/adma.202504355. PMC12535766 40878551

[31] S. im Sande , Y. Deng , S. I. Bozhevolnyi , and F. Ding , “Spin‐Controlled Generation of a Complete Polarization Set With randomly‐interleaved Plasmonic Metasurfaces,” Opto‐Electronic Advances 7, no. 8 (2024): 240076, 10.29026/oea.2024.240076.

[32] A. Wang and Y. Dan , “Mid‐Infrared Plasmonic Multispectral Filters,” Scientific Reports 8, no. 1 (2018): 11257, 10.1038/s41598-018-29177-0. PMC6062579 30050145

[33] H. Tan , J. Meng , and K. B. Crozier , “Multianalyte Detection With Metasurface‐Based Midinfrared Microspectrometer,” ACS Sensors 9, no. 11 (2024): 5839–5847, 10.1021/acssensors.4c01220. 39475063

[34] F. Kuruoglu , S. Rosas , Y. Chen , et al., “Dynamically Tunable Membrane Metasurfaces for Infrared Spectroscopy and Strong Light‐Matter Interactions,” Light: Science & Applications 15, no. 1 (2026): 269, 10.1038/s41377-026-02382-7. PMC13249898 42265082

[35] S. B. Sulejman , L. Wesemann , M. McCormack , et al., “Metasurfaces for Infrared Multimodal Microscopy: Phase Contrast and Bright Field,” ACS Photonics 12, no. 3 (2025): 1494–1506, 10.1021/acsphotonics.4c02097.

[36] X. Nan , Q. Zheng , Y. Dong , et al., “Near‐Infrared Imaging Highly Enhanced by Pixel‐Level Integrated Plasmonic Metasurfaces on CMOS Image Sensors,” Advanced Optical Materials 12, no. 35 (2024): 2401824, 10.1002/adom.202401824.

[37] D. E. Jung , V. J. Einck , A. Dawicki , et al., “Full Wafer Scale Manufacturing of Directly Printed TiO_2_ Metalenses at Visible Wavelengths With Outstanding Focusing Efficiencies,” Advanced Materials 37, no. 30 (2025): 2500327, 10.1002/adma.202500327. PMC12306388 40342182

[38] M. C. Traub , W. Longsine , and V. N. Truskett , “Advances in Nanoimprint Lithography,” Annual Review of Chemical and Biomolecular Engineering 7, no. 1 (2016): 583–604, 10.1146/annurev-chembioeng-080615-034635. 27070763

[39] C. Park , W. Kim , Y. Kim , et al., “High‐Throughput Fabrication of Large‐Scale Metaholograms via One‐Step Printing,” Advanced Optical Materials 12, no. 9 (2024): 2301562, 10.1002/adom.202301562.

[40] A. McClung , M. Torfeh , V. J. Einck , J. J. Watkins , and A. Arbabi , “Visible Metalenses With High Focusing Efficiency Fabricated Using Nanoimprint Lithography,” Advanced Optical Materials 12, no. 9 (2024): 2301865, 10.1002/adom.202301865.

[41] J. Schmidt‐Hieber , “Nonparametric Regression Using Deep Neural Networks With ReLU Activation Function,” Annals of Statistics 48, no. 4 (2020): 1875–1897, 10.1214/19-aos1875.

[42] M. Rocha , P. Cortez , and J. Neves , “Evolution of Neural Networks for Classification and Regression,” Neurocomputing 70, no. 16–18 (2007): 2809–2816, 10.1016/j.neucom.2006.05.023.

[43] M. Bataineh and T. Marler , “Neural Network for Regression Problems With Reduced Training Sets,” Neural Networks 95 (2017): 1–9, 10.1016/j.neunet.2017.07.018. 28843090

[44] J. Xu , A. Wang , and Y. Dan , “Plasmonic Micropipe Spectral Filters in Mid‐infrared,” Optics Letters 44, no. 18 (2019): 4479–4482, 10.1364/ol.44.004479. 31517911

[45] M. N. Julian , C. Williams , S. Borg , S. Bartram , and H. J. Kim , “Reversible Optical Tuning of GeSbTe Phase‐Change Metasurface Spectral Filters for Mid‐wave Infrared Imaging,” Optica 7, no. 7 (2020): 746–754, 10.1364/optica.392878. PMC8262593 34277892

[46] A. Soliman , C. Williams , R. Hopper , F. Udrea , H. Butt , and T. D. Wilkinson , “High‐Transmission Mid‐infrared Bandpass Filters Using Hybrid Metal‐Dielectric Metasurfaces for CO_2_ Sensing,” Advanced Optical Materials 13, no. 8 (2025): 2402603, 10.1002/adom.202402603.

[47] M. Debenham , “Refractive Indices of Zinc Sulfide in the 0.405–13‐μm Wavelength Range,” Applied Optics 23, no. 14 (1984): 2238–2239, 10.1364/ao.23.002238. 18212982

