# Supplementary material for: Nanoimprinted Infrared Metasurface Multispectral Filters for Neural‐Network‐Assisted Low‐Temperature Sensing
Source: Nanophotonics. 2026 Nov 2;15(21):e70345. doi: 10.1002/nap2.70345 (PMC13726500; doi:10.1002/nap2.70345)
Supplement: Supplementary file 1 [file NAP2-15-e70345-s001.pdf]

Supporting Information

Nanoimprinted Infrared Metasurface Multispectral Filters for Neural-Network-Assisted Low-Temperature Sensing

Shiyu Yang, Zhijuan Su*, and Yaping Dan*

Manuscript 7857234

S1 Fabrication and structural evidence

The 8-inch silicon master is patterned using a Canon FPA-3030EX6 deep-ultraviolet (DUV) stepper at 248 nm, followed by silicon etching. Step-and-repeat exposure expands the periodic ring and pixel layouts from a compact mask field across the master. The stepper positioning precision is approximately 30 nm. The Eitre-6 nanoimprint system uses 6-inch circular intermediate polymer stamp (IPS) films; the 8-inch master is divided into four sectors for loading in the available 6-inch system. This division is determined by the equipment loading size. The patterned silicon-master sector is first replicated onto the 6-inch IPS film, whose relatively rigid patterned surface helps retain the ring relief. This working stamp then copies the required patterned area to the resist-coated 1-inch ZnS substrate. Each 1-inch ZnS device receives one full-area imprint, transferring the periodic layout established on the master. Imprinting uses 65–75 °C, 10–20 bar and a 120 s holding time. Exposure at 365 nm and 40 mW cm^{-2} for 20 s supplies approximately 800 mJ cm^{-2}.

The fabrication route is chosen primarily to combine fine-feature patterning with practical large-area fabrication on ZnS. Conventional contact lithography is difficult for the submicrometer ring features, whereas electron-beam lithography can resolve them but requires time-consuming serial writing over the device area. DUV stepper lithography defines these features on a reusable silicon master. The available stepper is configured for large silicon wafers rather than the small ZnS optical substrates used here. Nanoimprinting therefore transfers the silicon-master pattern onto a 1-inch ZnS substrate, matching the detector window without requiring a costly larger ZnS substrate. Repeated stepper exposures extend a compact mask pattern across the master; the completed pattern is then copied in one imprint. Master reuse also supports manufacture of additional sensors, even when each sensor has a long service life.

Devices use 1-inch, 1-mm-thick double-side-polished ZnS substrates, cleaned in acetone, isopropanol and deionized water for 3 min each. A Denton Explorer-14 electron-beam evaporator deposits 3 nm Cr and 100 nm Au at 0.1 and 0.5 Å s^{-1}, respectively, with a base pressure of approximately 1×10^{-6} Torr; Au purity is 99.999%. Nanoimprinting uses TU7-220 resist and an IPS working stamp. After metal pattern transfer and resist removal, a 300 nm ZnS overlayer is deposited at 3 Å s^{-1}.

The SENTECH SI 500 plasma recipe for silicon etching uses CHF_3/Ar = 24/30 sccm, 0.8 Pa, 450 W inductively coupled plasma power, 100 W radio-frequency power and a 15 °C electrode temperature, with a programmed duration of 86 s. Residual-layer clearance uses Cl_2/Ar = 30/20 sccm, 0.5 Pa, 450/100 W and 20 °C, with a programmed duration of 32 s and the reported 20% overetch allowance. Ar ion-beam pattern transfer then uses 550 eV ions, a 65 mA beam current and normal incidence for 130 s, including 30 s of overetching. The remaining resist is stripped after ion-beam etching using the same Cl_2/Ar recipe.

Each device receives one imprint, so resist at the stamp perimeter does not need to be cleared for another overlapping imprint. Within the patterned area, the residual resist layer is removed before Ar ion-beam transfer to expose the metal where the apertures are formed. The approximately 30 nm placement precision refers to positioning the successive DUV exposure fields on the silicon master.

S2 Spectral characterization and sampled-position statistics

Spectra were acquired with a Thermo Scientific iN10 MX microscope using bare ZnS as the reference. The available measurement record reports a 25 μm spot, approximately 12 s acquisition and 0.4 cm^{-1} resolution. Let R(λ) denote the measured transmission signal of the patterned filter divided by that of bare ZnS. The reported transmittance is $T(\lambda) = R(\lambda) \times \tau$, where $\tau \approx 0.85$ is the adopted single-interface ZnS transmission factor (bare-substrate transmission is approximately 0.70). This correction was applied once. It expresses the filter response on the convention used for comparison with the ZnS-substrate simulation, which excludes the rear substrate–air interface; it is distinct from the total air-to-air transmission of the finite-thickness device. For each design, five spectra are labeled center, upper, lower, left and right. Peak wavelength and transmittance are determined from the exported spectrum maximum. Full width at half maximum is the distance between the nearest interpolated half-peak crossings, with no baseline subtraction. Sample standard deviation uses $n-1$ and describes spatial variation within the five sampled locations.

Figure S1 compares simulated spectra with representative measured spectra for the four filter designs. Figure S2 separately examines spatial variation using an additional measurement set with five sampled positions per design. Table S1 reports the representative spectra, while Table S2 summarizes the five-position set; the two summaries are calculated separately.

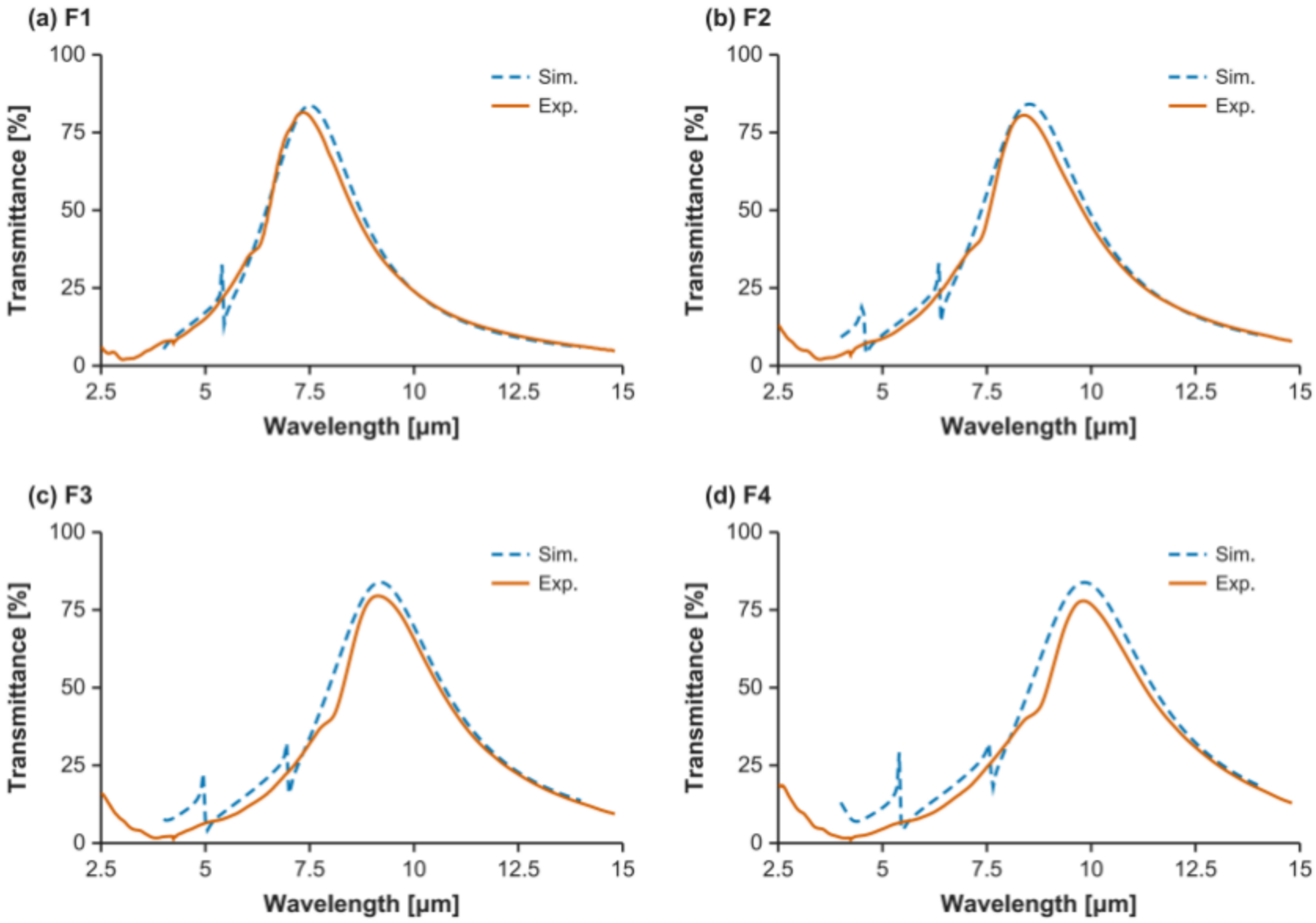

Figure S1. Direct simulation/measurement comparison for filters 1–4. The measured curves retain the supplied single-interface correction convention. Simulated and measured spectra are not shifted or amplitude-scaled to improve agreement. Table S1 gives the exported maxima and half-peak widths.

Table S1. Comparison of simulated and measured spectral peaks and full widths at half maximum (FWHM).

Filter	λ sim / exp [μm]	Peak sim / exp [%]	FWHM sim / exp [μm]
1	7.500 / 7.325	83.67 / 81.48	2.742 / 2.555
2	8.500 / 8.390	84.15 / 80.58	3.103 / 2.898
3	9.200 / 9.130	83.86 / 79.51	3.358 / 3.088
4	9.850 / 9.820	83.86 / 77.91	3.587 / 3.537

Figure S1 and Table S1 compare simulation and measurement on the same wavelength and transmission scales. The measured peaks occur at 7.325, 8.390, 9.130 and 9.820 μm, blue-shifted by 0.175, 0.110, 0.070 and 0.030 μm from simulation; their peak transmission decreases by 2.19, 3.57, 4.35 and 5.95 percentage points. Measured FWHMs are 2.555, 2.898, 3.088 and 3.537 μm, compared with simulated values of 2.742, 3.103, 3.358 and 3.587 μm. The finished-device FIB cross section (Figure S3) shows sloped aperture walls and an outer lateral span of 1.668 μm, compared with the designed outer diameter of 1.7 μm. Sloped walls change the opening size through the film thickness and can therefore change the resonance wavelength; a smaller effective opening can favor a shift toward shorter wavelengths. The simulation assumes a flat ZnS top surface, whereas the deposited ZnS follows the underlying steps. This changes the shape and distribution of ZnS around the apertures and can change the resonance wavelength, peak transmission and bandwidth. Deposited-film roughness and differences in optical constants can also change absorption and the amount of transmitted light. Together, these structural and optical differences provide specific physical explanations for the observed spectral differences. The measured filters retain the broad, overlapping passbands needed for thermal spectral encoding. The fabricated stack contains a 3 nm Cr adhesion layer. ZnS dispersion uses the Debenham dataset [1] within its reported 0.405–13 μm range, with a formula extension outside that range. The experimental spectra use the single-interface transmission correction defined above.

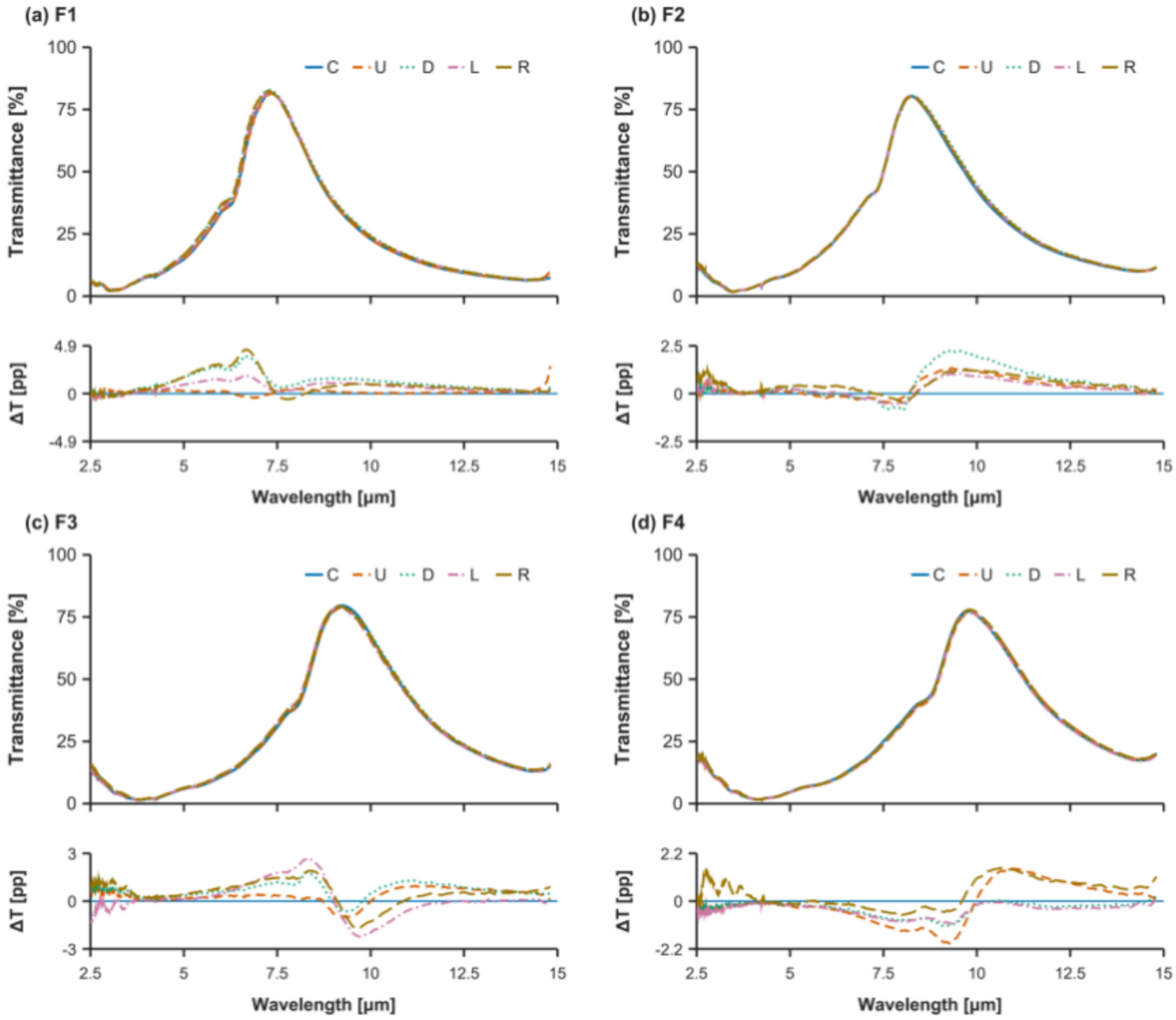

Figure S2. Spectra at five sampled locations for each filter design. Internal keys and line styles identify C: center; U: upper; D: lower; L: left; R: right. The lower plot in each panel gives $\Delta T = T_{position} - T_{center}$ in percentage points (pp), calculated pointwise on the measured wavelength grid.

Table S2. Mean ± sample SD across five sampled positions per design. These statistics describe the spatial variation of the sampled filter spectra.

**Filter**	**n**	**Peak λ [µm]**	**Peak T [%]**	**FWHM [µm]**
1	5	7.320 ± 0.042	81.97 ± 0.55	2.501 ± 0.042
2	5	8.255 ± 0.018	80.30 ± 0.20	3.042 ± 0.035
3	5	9.193 ± 0.033	79.08 ± 0.38	3.227 ± 0.064
4	5	9.792 ± 0.016	77.18 ± 0.47	3.532 ± 0.031

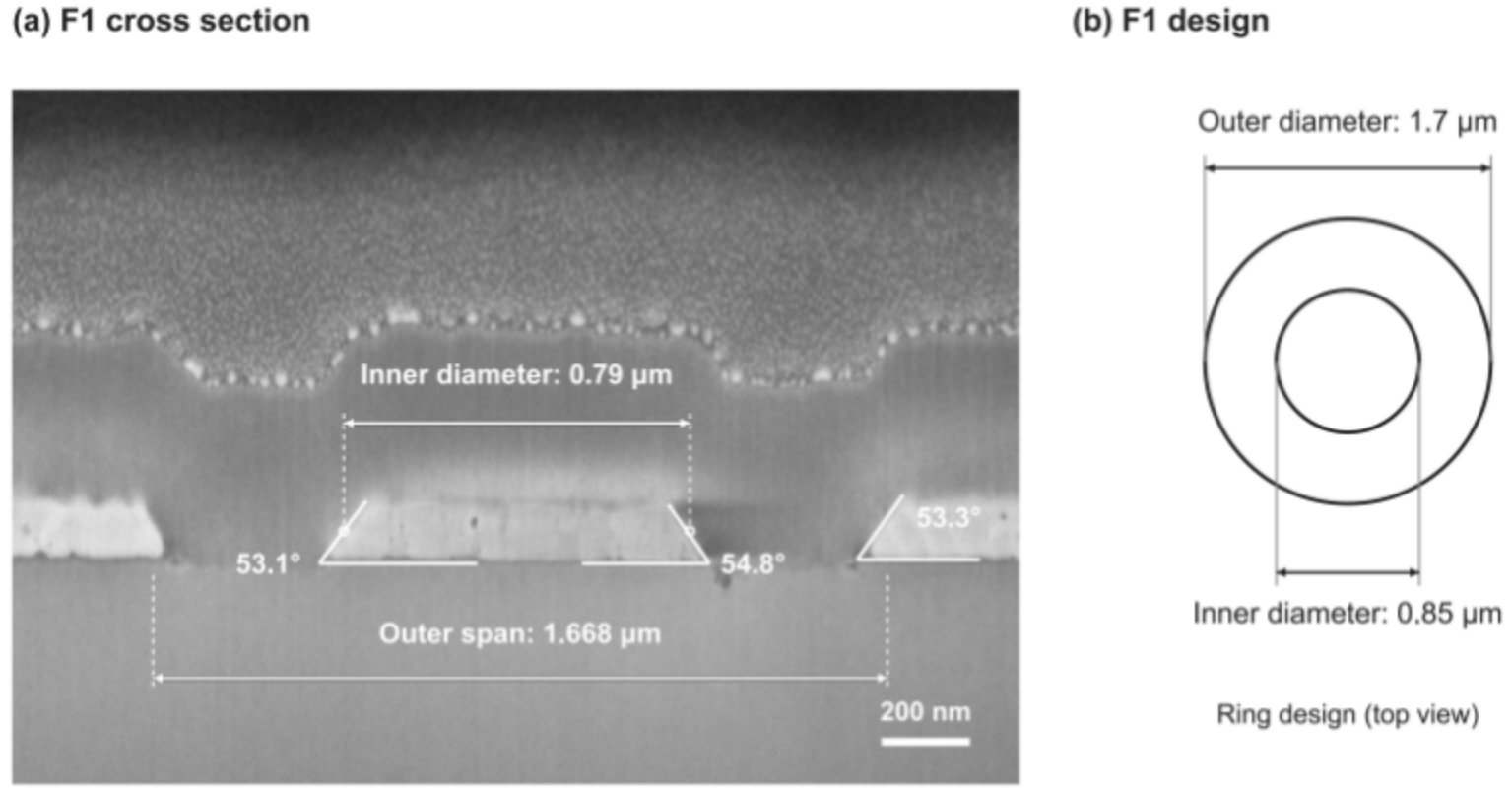

Figure S3. (a) FIB cross section of filter design 1 along the ring-diameter direction. The local profile angles are 53.1°, 54.8° and 53.3°. The outer span is 1.668 µm; the inner diameter measured at the metal-layer mid-height is approximately 0.79 µm. Scale bar: 200 nm. (b) Ring design with an outer diameter of 1.7 µm and an inner diameter of 0.85 µm. The cross-sectional dimensions refer to the horizontal spans marked in (a).

Table S3. Comparison of designed ring diameters and FIB cross-sectional dimensions.

Parameter	Design [µm]	FIB cross section [µm]
Outer diameter	1.7	1.668
Inner diameter	0.85	0.79

The annotated outer span is 1.668 µm, compared with the designed outer diameter of 1.7 µm, a difference of 0.032 µm (1.9%). The inner diameter at the metal-layer mid-height is approximately 0.79 µm, compared with the designed 0.85 µm, a difference of approximately 0.06 µm (7%). The inner span is read between the two sidewall boundaries at the height marked in Figure S3a, using the calibrated horizontal pixel size of 2.233 nm. This measurement position makes the comparison well defined for the sloped profile.

S3 Temperature measurements and regression outputs

The blackbody source model is SC-LM100-L-4A/4D. Its exit is approximately 19 cm from the filter wheel, which is approximately 1 cm in front of the Dewar window. The 1-inch filter size matches the approximately 1-inch detector optical-window size. The complete optical test system is inside a nitrogen-filled low-temperature chamber with an ambient setpoint of −40 °C to suppress frosting. The liquid-nitrogen-cooled infrared linear-array detector with a cold-shield tube between the optical window and the array, aligned along their common optical axis, is read with a 4200 source-measure instrument at a fixed bias of 0.2 V. At each blackbody setpoint, four single-pattern filters and unfiltered wheel positions are read sequentially; the filtered readout averages four pixels. The blackbody temperature is scanned from −20 to 20 °C in 0.1 °C steps, with readings taken after current fluctuation falls below the 1% stability criterion. Four separately measured unfiltered pixel currents supply the four-unfiltered-input control. The optical configuration tests spectral temperature encoding without the bonding and packaging required for direct two-dimensional focal-plane integration.

The calibration curves are processed in Origin using averaging, Savitzky–Golay smoothing (reported window: 200 points; polynomial order: 2) and interpolation onto a uniform 0.01 °C grid of 4001 points. The mean blackbody response changes smoothly with temperature in this fixed optical configuration. Averaging reduces readout fluctuations. The quadratic Savitzky–Golay fit allows local curvature without

using a high-order polynomial to follow individual fluctuations. Smoothing is applied before interpolation to the original measurements sampled at 0.1 °C intervals. The 200-point window spans approximately 20 °C and provides a broad neighborhood for estimating the slowly varying calibration trend. Interpolation follows this smoothing step. Interpolation aligns all channels on a common grid for regression; it does not add independently measured temperatures or establish a 0.01 °C instrumental resolution. The workflow fits input and target StandardScaler objects on the full processed dataset before randomly assigning 2800, 600 and 601 points to training, validation and testing. Accordingly, the test evaluation includes full-dataset normalization information and neighboring processed-curve points. It characterizes regression on the processed calibration curves. All six configurations use identical test-target rows. MAE and RMSE are calculated from prediction minus blackbody target temperature; target-dependent MAE is calculated in fixed 5 °C bins using all 601 test points. Table S5 states the interval boundaries and counts.

The neural-network regressor uses fully connected layers m→256→128→64→32→16→1, batch normalization in the first two hidden blocks, LeakyReLU activations, dropout 0.15 in the first two blocks and 0.10 in the third, AdamW optimization with learning rate 0.002 and weight decay 10^{-5}, mean-squared-error loss and batch size 64. The maximum training length is 300 epochs; validation-based early stopping has patience 40. A ReduceLROnPlateau scheduler uses factor 0.7 and patience 15. NumPy and PyTorch seeds are set to 42.

These choices match a calibration task with a few current inputs and one continuous temperature output. Fully connected layers combine all input channels, and LeakyReLU provides nonlinear mapping while retaining gradients for negative activations. Input and target standardization support numerical optimization; batch normalization stabilizes intermediate activation scales. Dropout and weight decay regularize the fit so that it is less dependent on particular hidden units or large weights.

Mean-squared-error loss gives a larger penalty to larger temperature deviations; MAE is reported separately in degrees Celsius. Minibatches of 64 are used for parameter updates. The scheduler reduces the initial learning rate of 0.002 by a factor of 0.7 when validation loss plateaus, with patience 15. The longer early-stopping patience of 40 permits continued optimization after a learning-rate reduction, within a maximum budget of 300 epochs. The input configurations use the same architecture rule and training settings, changing only the input dimension.

All six input configurations are evaluated on 601 unique, identically ordered reference temperatures between −20 and 20 °C. Metrics use $e = \text{prediction} - \text{target}$, $\text{MAE} = \text{mean}(|e|)$, $\text{RMSE} = \text{sqrt}(\text{mean}(e^2))$ and $R^2 = 1 - \Sigma e^2/\Sigma(\text{target} - \text{mean}(\text{target}))^2$. Target temperatures refer to blackbody setpoints; the reported MAE evaluates regression relative to those setpoints rather than an independently calibrated absolute temperature uncertainty. A filtered feature averages four detector pixels, whereas the four-unfiltered configuration uses four separately measured pixel currents as four features. The identical target rows and stated training settings define the regression comparison; the acquisition configurations retain these pixel-averaging differences.

Table S4. Regression metrics for six input configurations evaluated on the same 601 target temperatures. The two-filter configuration is F1+F2.

Configuration	n	MAE [°C]	RMSE [°C]	Maximum |e| [°C]
Unfiltered (1)	601	0.433857	0.518941	1.217940
Unfiltered (4)	601	0.713403	0.915997	2.047007
F1	601	0.518406	0.865751	3.622984
F1+F2	601	0.341123	0.404921	0.949959
F1+F2+F3	601	0.254986	0.281055	0.671982
F1+F2+F3+F4	601	0.160217	0.208988	0.585770

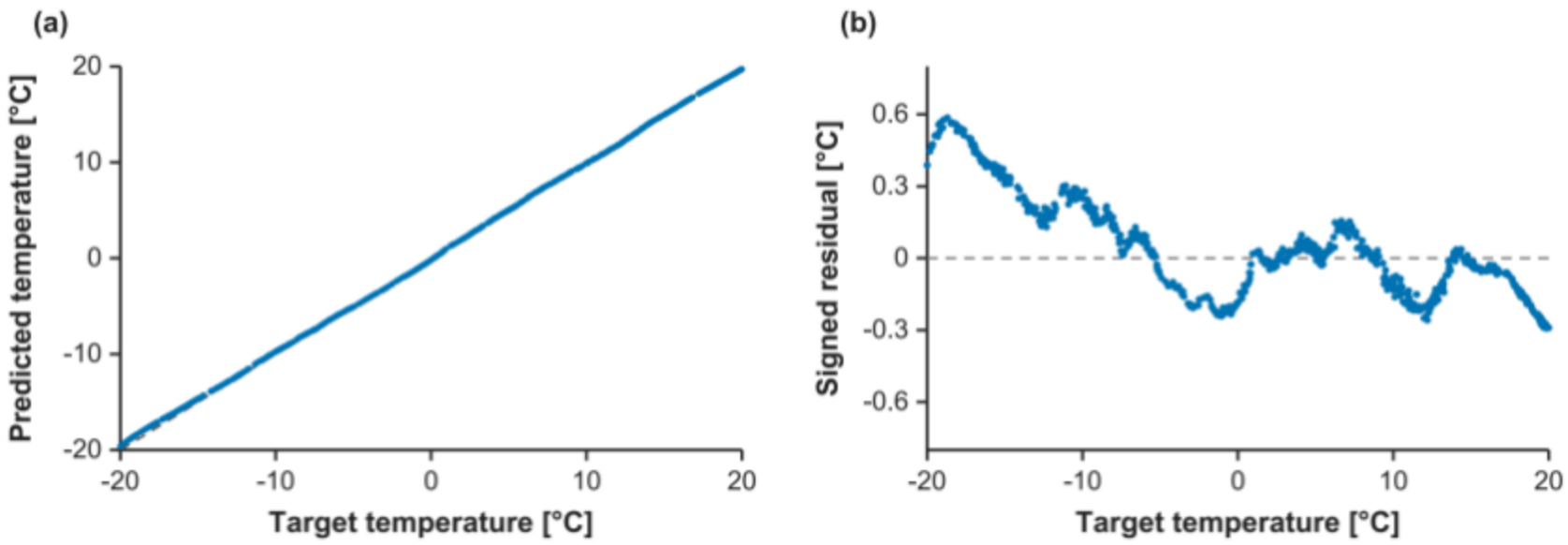

Figure S4. (a) Four-filter predicted temperatures versus blackbody target temperatures; dashed line: equality. (b) Signed residuals for the same 601 exported pairs. These processed-test-grid statistics accompany the original Figure 5c error histogram; no independent-scan accuracy is inferred.

Table S5. Target-temperature-dependent MAE in °C for fixed 5 °C intervals. n counts processed test-grid points and totals 601 for each configuration. Each value averages errors over target temperatures within the indicated interval.

**Interval [°C]**	**n**	**U1 MAE**	**U4 MAE**	**Four-filter MAE**
[-20, -15)	66	0.9738	1.9268	0.4548
[-15, -10)	66	0.8010	1.3181	0.2315
[-10, -5)	78	0.5434	0.7229	0.1103
[-5, 0)	62	0.2162	0.0920	0.1898
[0, 5)	82	0.2243	0.2001	0.0508
[5, 10)	76	0.3500	0.3020	0.0605
[10, 15)	86	0.1974	0.4526	0.1256
[15, 20]	85	0.3042	0.8731	0.1300

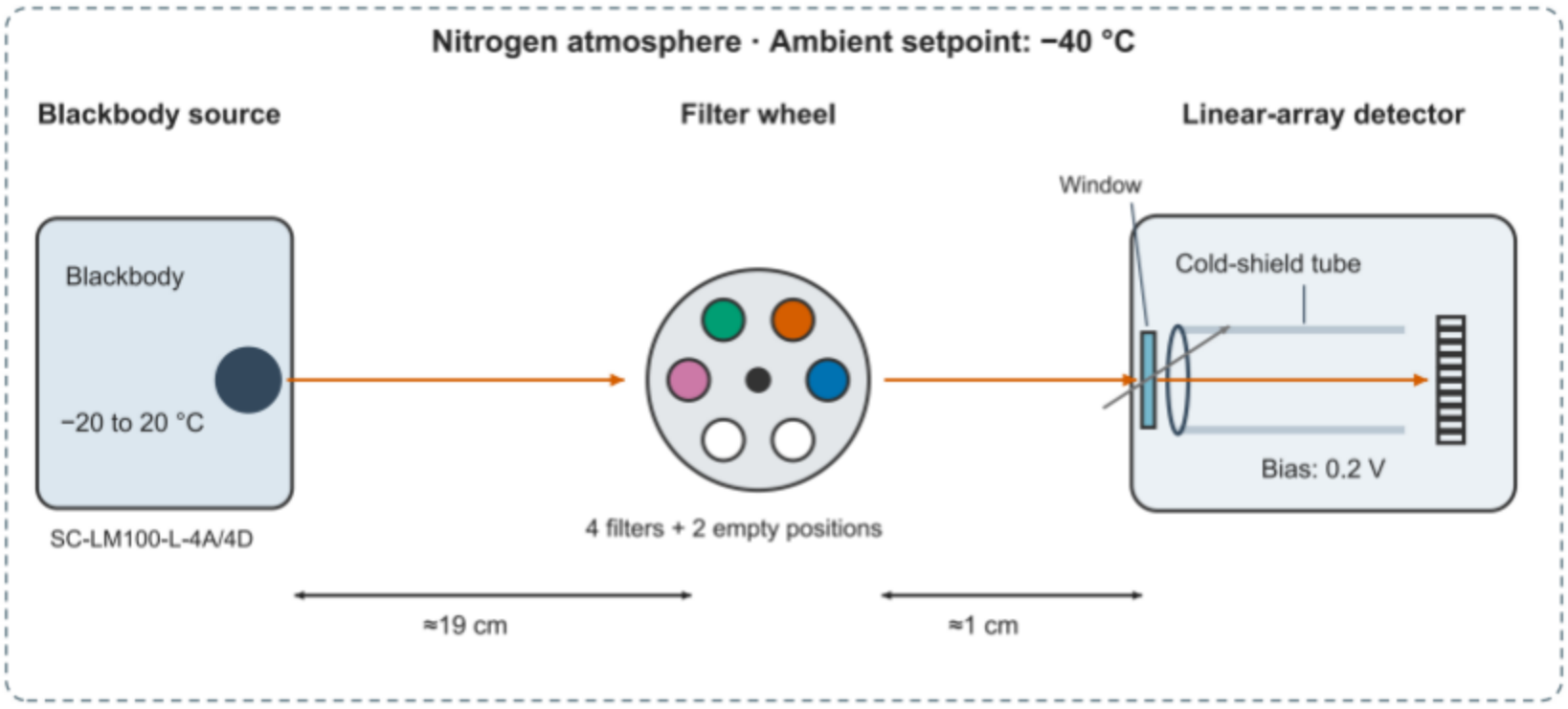

Figure S5. Experimental optical path. Four single-pattern filters and two empty positions are switched manually at each blackbody target temperature. The target is scanned in 0.1 °C steps from −20 to 20 °C. Blackbody-to-filter and filter-to-window separations are approximately 19 cm and 1 cm. The detector is liquid-nitrogen cooled and biased at 0.2 V. The dashed boundary encloses the complete optical test system in a nitrogen atmosphere at an ambient setpoint of −40 °C. The cold shield is shown as an internal sleeve between the optical window and the linear array. The sleeve axis, window center and line-array center are aligned horizontally; the sleeve restricts the accepted angles and suppresses off-axis stray radiation. The sleeve dimensions and illustrative ray are schematic.

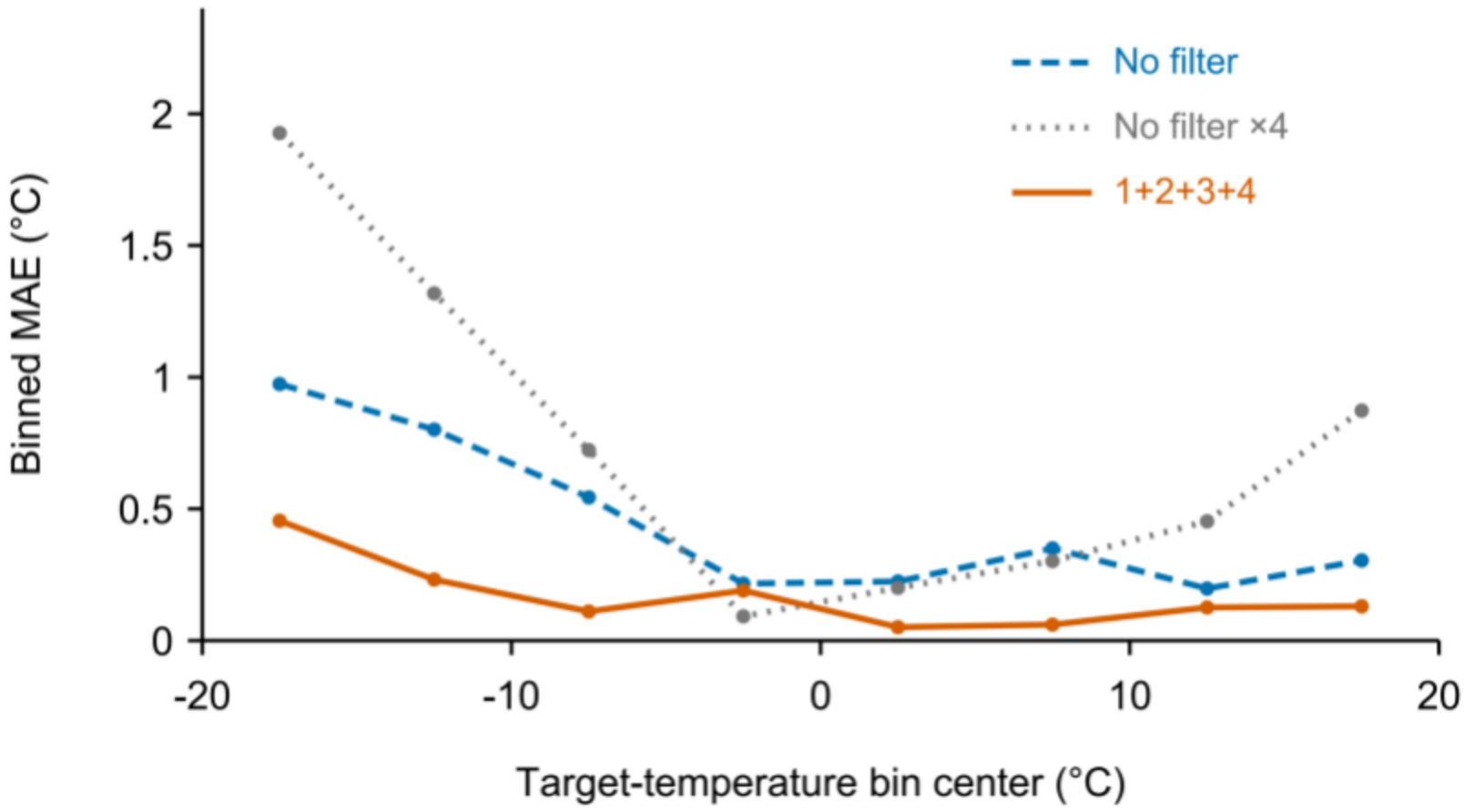

Figure S6. Target-temperature-dependent MAE for one unfiltered input, four unfiltered pixel inputs and four filtered inputs. Errors are averaged within fixed 5 °C intervals; points are placed at the interval centers. Table S5 lists all eight intervals and their counts, totaling 601 processed test-grid points per configuration. The overall six-configuration MAE comparison is shown in Figure 5b. The four-filter benefit varies with temperature and is greatest in the coldest interval.

S4 Local mathematical description of channel information

Temperature precision depends on the change in current per degree relative to the current noise. Let $I = \mu(T) + \eta$, where $\mu(T)$ is the mean current vector, $g = d\mu/dT$ contains its slopes, and the noise covariance Σ describes channel variances and correlations. For a local Gaussian model with temperature-independent covariance, the scalar-temperature Fisher information is $J(T) = g^T\Sigma^{-1}g$. For a locally unbiased estimator, $\mathrm{var}(T) \geq 1/J(T)$. This gives a conditional variance bound. If the local error is approximately zero-mean Gaussian, $\mathrm{MAE} \approx \sqrt{(2/\pi)} \times \mathrm{SD}$ relates mean absolute error to standard deviation. The empirical MAEs in Tables S4/S5 are calculated directly by averaging the absolute prediction-minus-target errors.

For m equal-slope channels with noise variance σ^2 and pairwise correlation ρ, $J_m = m\, g_0^2/[\sigma^2(1 + (m - 1)\rho)]$ and the variance bound is $\sigma^2[(1 - \rho)/m + \rho]/g_0^2$. Independent noise ($\rho = 0$) gives a $1/\sqrt{m}$ standard-deviation scaling. For positive correlation, the independent-noise contribution decreases with m, while the common-noise term $\sigma^2\rho/g_0^2$ remains. In the limit $\rho \rightarrow 1$, identical channel responses carry no additional independent information. Different spectral responses can supply complementary temperature slopes, whereas fixed affine copies of a processed current become redundant after standardization. In practice, the noise correlations, light available per channel and calibration stability limit the benefit of adding channels.

With nuisance parameters q, define $G = [\partial\mu/\partial T, \partial\mu/\partial q]$ and $F = G^T\Sigma^{-1}G$. When the nuisance block is invertible, effective temperature information is $J_{eff} = F_{TT} - F_{Tq}\, F_{qq}^{-1}\, F_{qT}$. Multiple channels help separate temperature from a nuisance only when their response derivatives are distinguishable. Figure S8 gives the relative detector spectral response that enters the channel weighting. A numerical information gain additionally requires the noise covariance and the relevant nuisance derivatives; the expressions here give the local mechanism and limiting conditions, while Tables S4 and S5 provide the empirical regression comparison.

The four-unfiltered control uses four separately measured pixel currents after the same reported processing. Identical target grids support a matched-target comparison of regression errors. The derivative/covariance

framework above explains how spectral response differences and noise correlation affect the information carried by multiple inputs; the reported MAEs quantify the present processed-curve regression evaluation.

S5 Context and measurement basis for the literature comparison

Table 1 compares measured peak transmittance for five experimental infrared-filter studies. Wang and Dan [2] report approximately 60% and reference spectra to bare Ge. Xu et al. [3] define local transmittance as transmitted intensity divided by the intensity incident on the measured array; their Figure 3a gives approximate peak levels of 25–60%. Tan et al. [4] report FTIR spectra of 41 patterned channels; Figure 2e gives peak levels around 40% as a graphical estimate. Julian et al. [5] report approximately 70% resonance transmission in the fabricated GST-embedded nanohole filters, measured by FTIR. Soliman et al. [6] report measured transmission above 60% using bare CaF_2 as reference. The present four spectra give peak transmittances of 77.91–81.48% under the single-interface ZnS correction convention. The selected studies establish representative throughput context across different infrared bands and structures, retaining each study's reference and measurement conditions rather than imposing a common renormalization. The tunable membranes [7] and phase-contrast microscopy metasurfaces [8] address narrow-resonance spectroscopy and polarization-dependent imaging, respectively; their relevance is discussed in the main text without using different application metrics as throughput values.

S6 Additional fabrication-stage images and detector response

Figure S7 illustrates DUV layout definition and nanoimprint transfer on representative process-test structures. The finished-device ring-diameter cross section used for optical interpretation is Figure S3. Figure S8 records the detector's relative spectral weighting over the filter band. Together, these observations complement the process conditions and the temperature-measurement configuration.

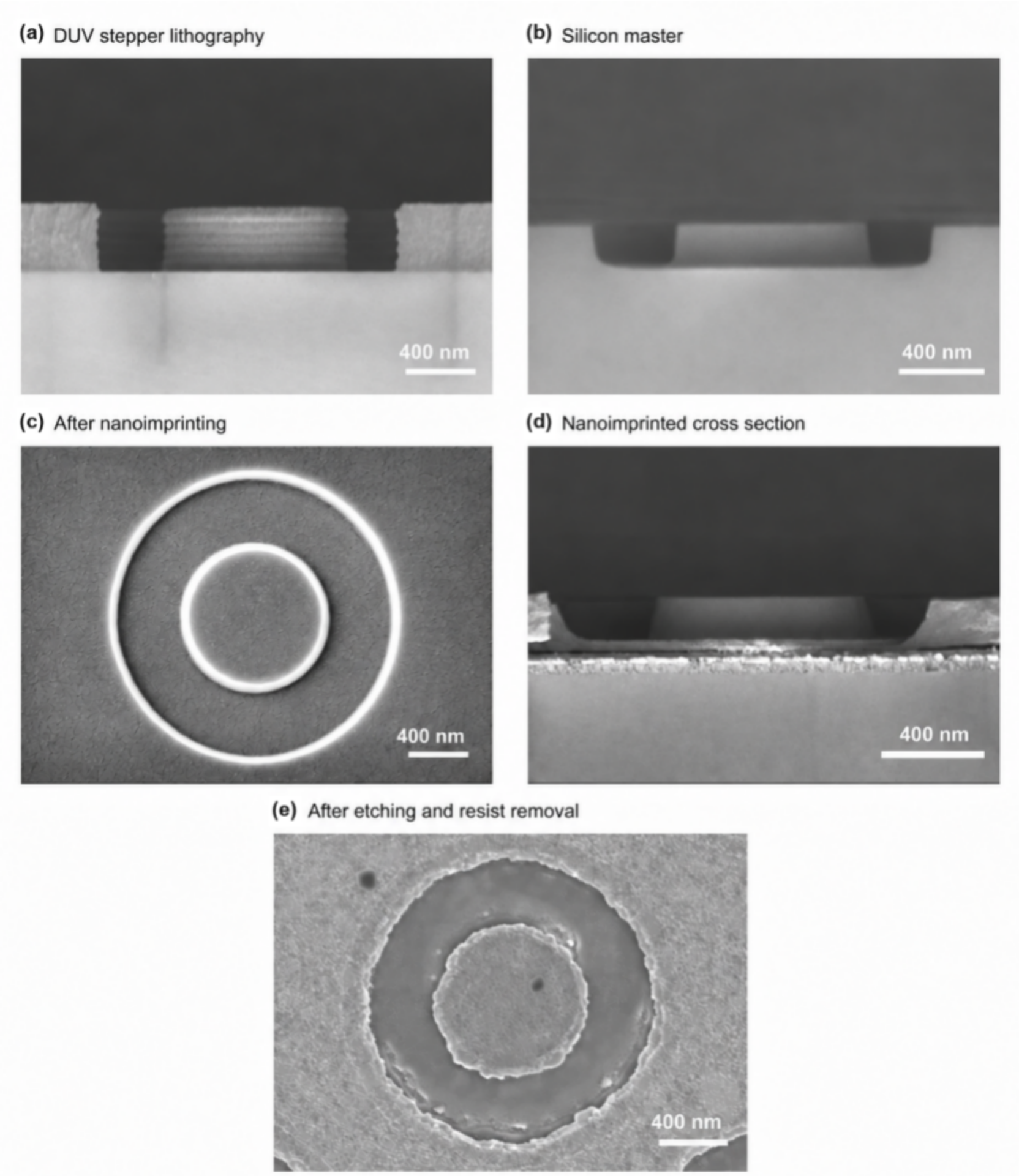

Figure S7. Representative SEM images at successive fabrication stages: (a) a cross section after DUV stepper lithography; (b) the silicon master; (c) a top view after nanoimprinting; (d) a cross section after nanoimprinting; and (e) a top view after etching and resist removal. The images illustrate pattern definition and transfer using representative process-test structures. Scale bars: 400 nm in all panels. The finished filter-design-1 cross section and design dimensions are shown in Figure S3.

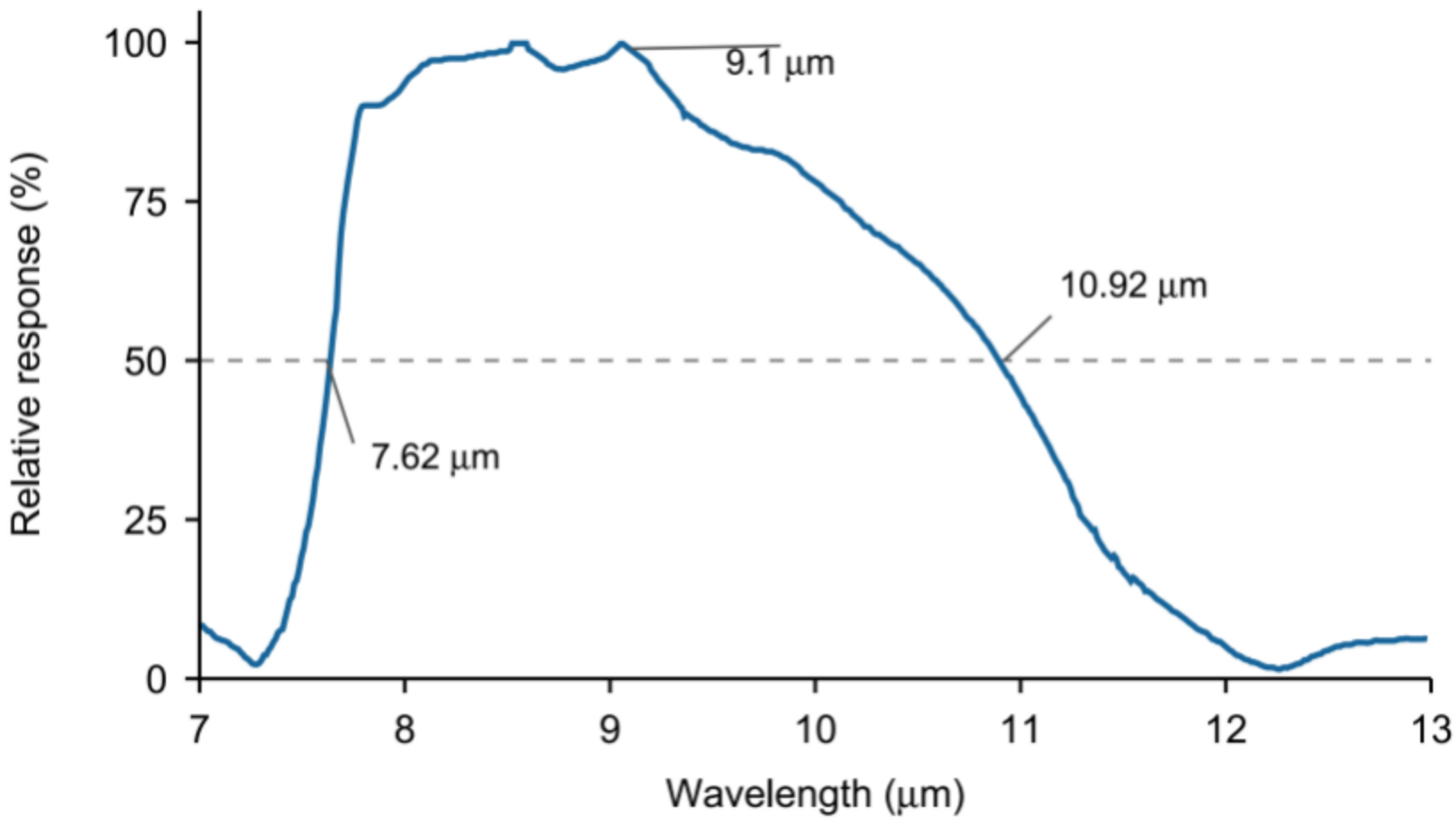

Figure S8. Relative spectral response of the detector used for the temperature measurements, redrawn by digitizing the recorded response plot. The original approximate annotations are retained: a peak near 9.1 μm and half-maximum wavelengths near 7.62 and 10.92 μm. The vertical axis gives relative response in percent on the original normalized scale. The curve illustrates the wavelength-dependent detector weighting in the optical chain.

S7 Spatial sampling and reconstruction for 2 × 2 multispectral readout

A four-band 2 × 2 mosaic assigns a different spectral band to each of four neighboring detector pixels. The four signals can then be combined into a multispectral temperature estimate. For a scene that is nearly uniform across a unit, this provides four spectral measurements of the same local temperature. At a boundary, spatial information is also needed to account for the different regions sampled by the four pixels. This is the same basic sampling issue encountered in an RGB Bayer camera: neighboring pixels record different bands, and reconstruction brings these measurements onto a common spatial grid.

The sampling trade-off can be stated directly. With detector pitch p, nonoverlapping 2 × 2 units give a temperature-sampling pitch of 2p in both directions and one quarter as many directly sampled temperature cells as a monochrome array of the same dimensions. For example, a 640 × 512 array contains 320 × 256 such units. Increasing the detector pixel count or reducing pixel pitch addresses this geometrical change. In particular, halving pixel pitch within the same image footprint gives four times as many pixels and restores the original temperature-sampling pitch. The optics and collected signal should be chosen together with pixel pitch to retain spatial detail and temperature precision.

Computational reconstruction complements denser sampling. After background and pixel-response calibration, demosaicing estimates each missing band from nearby pixels that measured that band. Edge-guided interpolation favors neighbors within the same region, reducing mixing across a boundary between warmer and cooler areas. Learned demosaicing also combines spatial features with correlations between spectral channels; Zhang et al. demonstrate this principle for multispectral filter-array images [9]. These methods can use information distributed across neighboring samples to improve reconstructed image detail.

Temperature imaging also permits joint spatial and temperature reconstruction. A model can search for a temperature map whose calibrated band responses agree with the measured mosaic currents, while using local spatial information to preserve boundaries. Filter-band selection, array layout and reconstruction should therefore be considered together, following the joint-design principle studied by Li et al. [10]. For infrared temperature readout, this is a reconstruction option based on the calibrated thermal responses. The precision–resolution balance can be evaluated with both temperature MAE and resolvable spatial detail, including boundary width and temperature error near edges as well as precision in uniform regions.

References

[1] M. Debenham, "Refractive indices of zinc sulfide in the 0.405–13-μm wavelength range," *Appl. Opt.*, vol. 23, no. 14, pp. 2238–2239, 1984.

[2] A. Wang and Y. Dan, "Mid-infrared plasmonic multispectral filters," *Sci. Rep.*, vol. 8, no. 1, Art. no. 11257, 2018.

[3] J. Xu, A. Wang, and Y. Dan, "Plasmonic micropipe spectral filters in mid-infrared," *Opt. Lett.*, vol. 44, no. 18, pp. 4479–4482, 2019.

[4] H. Tan, J. Meng, and K. B. Crozier, "Multianalyte detection with metasurface-based midinfrared microspectrometer," *ACS Sensors*, vol. 9, no. 11, pp. 5839–5847, 2024.

[5] M. N. Julian, C. Williams, S. Borg, S. Bartram, and H. J. Kim, "Reversible optical tuning of GeSbTe phase-change metasurface spectral filters for mid-wave infrared imaging," *Optica*, vol. 7, no. 7, pp. 746–754, 2020.

[6] A. Soliman, C. Williams, R. Hopper, F. Udrea, H. Butt, and T. D. Wilkinson, "High-transmission mid-infrared bandpass filters using hybrid metal-dielectric metasurfaces for CO_2 sensing," *Adv. Opt. Mater.*, vol. 13, no. 8, Art. no. 2402603, 2025.

[7] F. Kuruoglu *et al.*, "Dynamically tunable membrane metasurfaces for infrared spectroscopy and strong light-matter interactions," *Light Sci. Appl.*, vol. 15, no. 1, Art. no. 269, 2026.

[8] S. B. Sulejman *et al.*, "Metasurfaces for infrared multimodal microscopy: phase contrast and bright field," *ACS Photonics*, vol. 12, no. 3, pp. 1494–1506, 2025.

[9] X. Zhang, Y. Dai, G. Zhang, X. Zhang, and B. Hu, "A snapshot multi-spectral demosaicing method for multi-spectral filter array images based on channel attention network," *Sensors*, vol. 24, no. 3, Art. no. 943, 2024.

[10] K. Li, D. Dai, and L. Van Gool, "Jointly learning band selection and filter array design for hyperspectral imaging," in *Proc. IEEE/CVF Winter Conf. Appl. Comput. Vis. (WACV)*, 2023, pp. 6384 – 6394.
